# Polycomb Requires *Chaperonin Containing TCP-1 Subunit 7* for Maintaining Gene Silencing in *Drosophila*

**DOI:** 10.3389/fcell.2021.727972

**Published:** 2021-10-01

**Authors:** Najma Shaheen, Jawad Akhtar, Zain Umer, Muhammad Haider Farooq Khan, Mahnoor Hussain Bakhtiari, Murtaza Saleem, Amir Faisal, Muhammad Tariq

**Affiliations:** ^1^Epigenetics and Gene Regulation Laboratory, Department of Biology, Syed Babar Ali School of Science and Engineering, Lahore University of Management Sciences, Lahore, Pakistan; ^2^Department of Physics, Syed Babar Ali School of Science and Engineering, Lahore University of Management Sciences, Lahore, Pakistan; ^3^Cancer Therapeutics Laboratory, Department of Biology, Syed Babar Ali School of Science and Engineering, Lahore University of Management Sciences, Lahore, Pakistan

**Keywords:** Polycomb, trithorax, TRiC, CCT7, chaperonin, cell fates, epigenetic cellular memory

## Abstract

In metazoans, heritable states of cell type-specific gene expression patterns linked with specialization of various cell types constitute transcriptional cellular memory. Evolutionarily conserved Polycomb group (PcG) and trithorax group (trxG) proteins contribute to the transcriptional cellular memory by maintaining heritable patterns of repressed and active expression states, respectively. Although chromatin structure and modifications appear to play a fundamental role in maintenance of repression by PcG, the precise targeting mechanism and the specificity factors that bind PcG complexes to defined regions in chromosomes remain elusive. Here, we report a serendipitous discovery that uncovers an interplay between Polycomb (Pc) and chaperonin containing T-complex protein 1 (TCP-1) subunit 7 (CCT7) of TCP-1 ring complex (TRiC) chaperonin in *Drosophila*. CCT7 interacts with Pc at chromatin to maintain repressed states of homeotic and non-homeotic targets of PcG, which supports a strong genetic interaction observed between *Pc* and *CCT7* mutants. Depletion of CCT7 results in dissociation of Pc from chromatin and redistribution of an abundant amount of Pc in cytoplasm. We propose that CCT7 is an important modulator of Pc, which helps Pc recruitment at chromatin, and compromising CCT7 can directly influence an evolutionary conserved epigenetic network that supervises the appropriate cellular identities during development and homeostasis of an organism.

## Introduction

In multicellular eukaryotes, epigenetic factors play a pivotal role in cell fate determination visualized by the differential gene expression profiles established during the patterning process in early development. In particular, maintenance of transcriptional states of important regulators like the Hox genes ensures faithful propagation of determined states during cell proliferation (Cavalli and Paro, 1999; Beuchle et al., 2001). The Polycomb group (PcG) and trithorax group (trxG) proteins maintain the heritable patterns of repressed and active gene expression states, respectively. An evolutionarily conserved hallmark of PcG and trxG regulation is the establishment and maintenance of long-term developmental decisions. Both groups exert their functions by interacting with the histones and the transcription machinery and by generating heritable epigenetic marks on chromatin (Kassis et al., 2017; Brand et al., 2019).

Molecular and biochemical analyses in *Drosophila* have unraveled the composition of at least two different PcG complexes, i.e., PRC1 and PRC2, and many accessory factors. Silencing by the PcG complexes correlates with histone H3 lysine 27 tri-methylation (H3K27me3) (Cao et al., 2002; Czermin et al., 2002; Müller et al., 2002) and histone H2A lysine 118 ubiquitination (H2AK118ub) (Wang et al., 2004). On the other hand, trxG proteins, discovered as suppressors of PcG, act as anti-silencing factors (Klymenko and Jürg, 2004), and several of them are known to act by modifying local properties of chromatin. For example, TRX and ASH1 directly counteract repression by catalyzing tri-methylation of histone H3 lysine 4 (H3K4me3) and histone H3 lysine 36 (H3K36me3), respectively. Moreover, trxG is much more heterogeneous with respect to protein complexes that covalently modify chromatin, remodel the chromatin, and interact with general transcription machinery (Kassis et al., 2017). Molecular chaperones such as heat shock cognate 4 (Hsc4) (Mollaaghababa et al., 2001), heat shock protein 90 (Hsp90) (Tariq et al., 2009; Sawarkar et al., 2012), and immunophilins (Anderson et al., 2002; Nelson et al., 2006; Chen, 2011) are proposed to serve as additional factors for PcG/trxG to maintain heritable patterns of gene expression. For example, the *Hsc4* mutant flies exhibit *Pc* like phenotype, and Hsc4 was found to be part of the PcG multi-protein complex (Mollaaghababa et al., 2001). Besides Hsc4, a novel *Drosophila* J class chaperone (Droj2) was also found stably associated with Polyhomeotic, a PcG protein, in *Drosophila* (Wang and Brock, 2003). Additionally, mutations in Hsp90 mimic trxG-like behavior, and TRX requires Hsp90 for maintenance of gene activation in *Drosophila* (Tariq et al., 2009). Genome-wide binding profile of Hsp90 shows a massive overlap with TRX at chromatin. Notably, Hsp90 also contributes to paused RNA polymerase and facilitates transcriptional block by PcG (Sawarkar et al., 2012). The presence of RNA polymerase and general transcription factors at silent promoters (Breiling et al., 2001; Dellino et al., 2004; Klymenko and Jürg, 2004; Enderle et al., 2011) and the competition observed between PcG and trxG to regulate the gene expression suggest that PcG and trxG proteins associate with their target genes as dynamic complexes that are in balance with one another (Kuroda et al., 2020). However, the mechanisms and the factors responsible to shift the balance in favor of either PcG or trxG during their dynamic association with chromatin remain elusive.

Here, we report a serendipitous discovery that describes direct molecular interactions of *Drosophila* Pc with chaperonin containing T-complex protein 1 (TCP-1) subunit 7 (CCT7) of TCP-1 ring complex (TRiC), a class of chaperones also called chaperonins. The TRiC chaperonins are protein-folding machines composed of hetero-oligomeric, double-ringed, high-molecular weight, ATP-dependent chaperones involved in the folding and assembly of multi-protein complexes (Gestaut et al., 2019). The complex architecture and mechanism of action of TRiC allow it to chaperone essential proteins involved in diverse cellular processes such as cell cycle regulation (Won et al., 1998; Camasses et al., 2003; Kaisari et al., 2017), cytoskeletal organization (Sternlicht et al., 1993), organ size (Kim and Choi, 2019), and signal transduction pathways (Guenther et al., 2002; Wells et al., 2006; Roobol et al., 2014; Antonova et al., 2018; Banks et al., 2018). In our quest for novel regulators of trxG, we had discovered *Drosophila CCT7* and *CCT5* subunits of TRiC as top trxG candidates influencing luciferase reporter in an *ex vivo* genome-wide RNA interference (RNAi) screen (Umer et al., 2019). However, contrary to our earlier discovery of *CCT7* and *CCT5* as trxG candidates in the RNAi screen, further analysis revealed that both *CCT7* and *CCT5* mutants genetically interact with *Pc* and strongly enhance extra sex comb phenotype. Moreover, suppression of *trx* mutant phenotype by *CCT7* mutation and a strong reactivation of homeotic genes in homozygous *CCT7* mutants corroborate the PcG-like behavior of these TRiC subunits. Importantly, the presence of CCT7 together with Pc on polytene chromosomes and its association at PcG targets, as characterized by chromatin immunoprecipitation (ChIP), support our hypothesis that CCT7 indeed contributes to repression by PcG. Our results further demonstrate that depletion of CCT7 results in dissociation of Pc from the chromatin, which explains the reactivation of PcG targets upon *CCT7* knockdown. Notably, CCT7 was found to interact with Pc at the molecular level in *Drosophila* cells, and a substantial amount of Pc was found in the cytoplasm when CCT7 was depleted. Together, our data provide an affirmative evidence of the role of CCT7 in regulating PcG-mediated gene expression patterns.

## Results

### TCP-1 Ring Complex Genetically Interacts With Polycomb Group and Trithorax Group System

**FIGURE 1:**
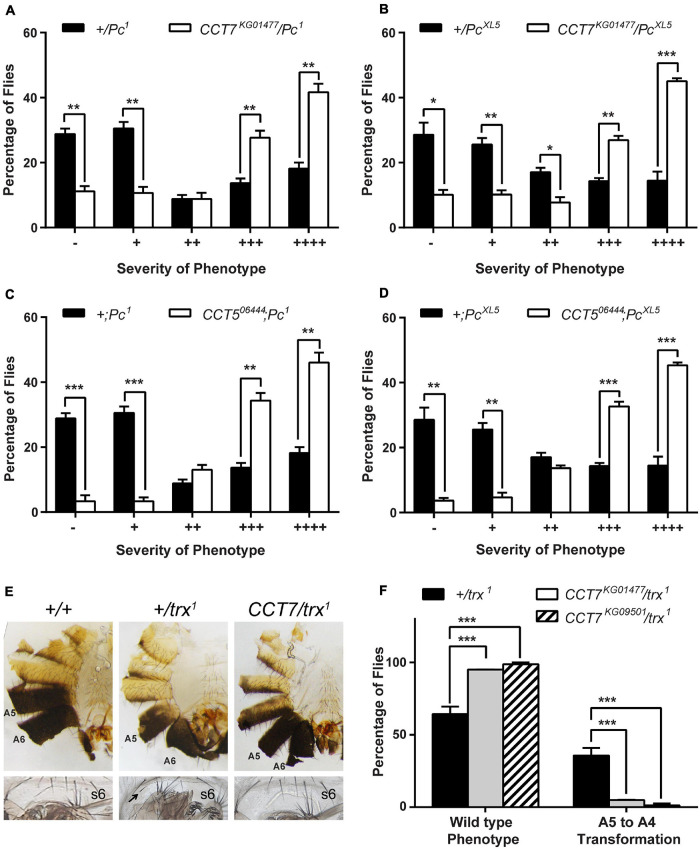
Mutants of TCP-1 ring complex (TRiC) exhibit Polycomb group (PcG)-like behavior. **(A–D)** Heterozygous male flies for *Pc* (*+/Pc^1^* and *+/Pc*^*XL5*^) obtained by crossing *Pc* (*Pc^1^* and *Pc*^*XL5*^) alleles with *w^1118^* exhibit strong extra sex comb phenotype, and these heterozygous males were used as control. *CCT7* (*CCT7*^*KG01477*^) and *CCT5* (*CCT5^06444^*) mutants crossed to two different alleles of *Pc* (*Pc^1^* and *Pc*^*XL5*^) significantly enhanced extra sex comb phenotype in *CCT7*^*KG01477*^*/Pc* **(A,B)** and *CCT5*^06444^*;Pc* **(C,D)** mutants, respectively, when compared with control (*+/Pc^1^* and *+/Pc*^*XL5*^). Here, 200 male flies with desired genotype from the progeny of each cross were scored for extra sex combs. Flies were categorized based on the number of extra sex comb bristles on the second and third pairs of legs (Tariq et al., 2009) as follows: –, no extra sex combs on second or third pair of legs; +, 1–2 bristles on the second leg; ++, 3 or more bristles on the second leg; +++, 3 or more bristles on the second leg and 1–2 bristles on the third leg; ++++, strong sex combs on both second and third pairs of legs. Percentages of flies for each category of phenotype, heterozygous *Pc^1^*/+ and *Pc*^*XL5*^/+ males and *CCT7*^*KG01477*^*/Pc*^1^ and *CCT7*^*KG01477*^*/Pc*^*XL5*^ double mutant male flies, are provided in Supplementary Table 1. The percentage of flies for each category was plotted as bar graphs. Error bars represent standard error of the mean (SEM) from three independent experiments. Statistical significance was calculated using *t*-test (* *p* ≤ 0.05, ** *p* ≤ 0.01, *** *p* ≤ 0.001, **** *p* ≤ 0.0001). **(E,F)** *CCT7/trx^1^* double mutant males from progeny of *CCT7* mutants crossed to *trx^1^* were scored for suppression of A5 to A4 transformation. As compared to wild-type flies (**E**, left panel), heterozygous *trx* mutant (*+/trx^1^*) males obtained from the cross of *w^1118^* with *trx^1^* mutant exhibit loss of abdominal pigmentation on A5 segment (**E**, middle panel), i.e., phenotype referred to as A5 to A4 transformation, and appearance of bristle on sternite 6 (s6, marked with black arrow). As compared to *trx* heterozygous (*+/trx^1^*) male flies, *CCT7* mutations suppressed A5 to A4 transformation phenotypes in *CCT7/trx^1^* double mutant male (**E**, right panel) flies. Flies were categorized into wild-type (**E**, left panel) and A5 to A4 transformation (**E**, middle panel) based on abdominal pigmentation phenotype. Percentage of flies obtained for each category of phenotype was plotted as bar graphs **(F)**. Error bars represent SEM from two independent experiments. Statistical significance was calculated using two-way ANOVA (*** *p* ≤ 0.001).

Since *CCT7* and *CCT5* subunits of TRiC chaperonin complex emerged as candidate trxG genes in a luciferase reporter-based genome-wide RNAi screen in *Drosophila* (Umer et al., 2019), we started validation of *CCT7* and *CCT5* as potential trxG genes. To investigate if TRiC subunits genetically interact with the PcG/trxG system, mutants for *CCT7* (*CCT7*^*KG*01477^ and *CCT7*^*KG0*9501^) and *CCT5* (*CCT5^06444^* and *CCT5*^*K06005*^) were crossed with two different *Pc* (*Pc^1^* and *Pc*^*XL5*^) alleles, and males from F1 progeny (*CCT7/Pc* and *CCT5/*+;*Pc/*+) were analyzed for extra sex comb phenotype. Heterozygous *Pc* (+*/Pc^1^* and +*/Pc*^*XL5*^) male flies, obtained from a cross of *Pc* (*Pc^1^* and *Pc*^*XL5*^) mutants with *w^1118^* mutant flies, exhibit strong extra sex comb phenotype. As compared to heterozygous *Pc* mutant males used as control, each mutant allele of *CCT7* and *CCT5* significantly enhanced extra sex comb phenotype of *Pc^1^* and *Pc*^*XL5*^ (Figures 1A–D and Supplementary Figures 1A–D). This strong genetic interaction of two different alleles of *CCT7* as well as *CCT5* with both the alleles of *Pc* indicates that *CCT7* and *CCT5* mutants behave like *PcG* genes. However, these results were contrary to the discovery of *CCT7* and *CCT5* as candidate trxG genes in the genome-wide RNAi screen (Umer et al., 2019). To further validate PcG-like behavior of TRiC subunits, it was investigated if *CCT7* mutants antagonize *trx* mutant phenotype, which is a hallmark of PcG genes. Both the mutant alleles of *CCT7* were crossed with *trx* (*trx^1^*) mutant to investigate if *CCT7* genetically interacts with *trx* and suppresses A5 to A4 homeotic phenotype of *trx* mutants. Heterozygous *trx* (*trx/*+) mutant males from the cross of *w^1118^* with *trx* exhibit loss of abdominal pigmentation (Figure 1E, middle), classified as A5 to A4 transformation (Ingham and Whittle, 1980). Both the alleles of *CCT7* strongly suppressed A5 to A4 transformation (Figure 1E, left) in *CCT7/trx* double mutants and resulted in a higher percentage of male flies with wild-type abdominal pigment (Figure 1F). A strong suppression of *trx* phenotype and enhancement of extra sex comb phenotype of *Pc* by both alleles of *CCT7* support the notion that *CCT7* acts as a potential PcG gene. Since luciferase is a known client of TRiC chaperonin (Dunn et al., 2001), it explains the presence of *CCT7* and *CCT5* subunits as trxG candidates in luciferase-based genome-wide RNAi screen.

### *Chaperonin Containing TCP-1 Subunit 7* Is Required for Polycomb Group-Mediated Gene Silencing

**FIGURE 2:**
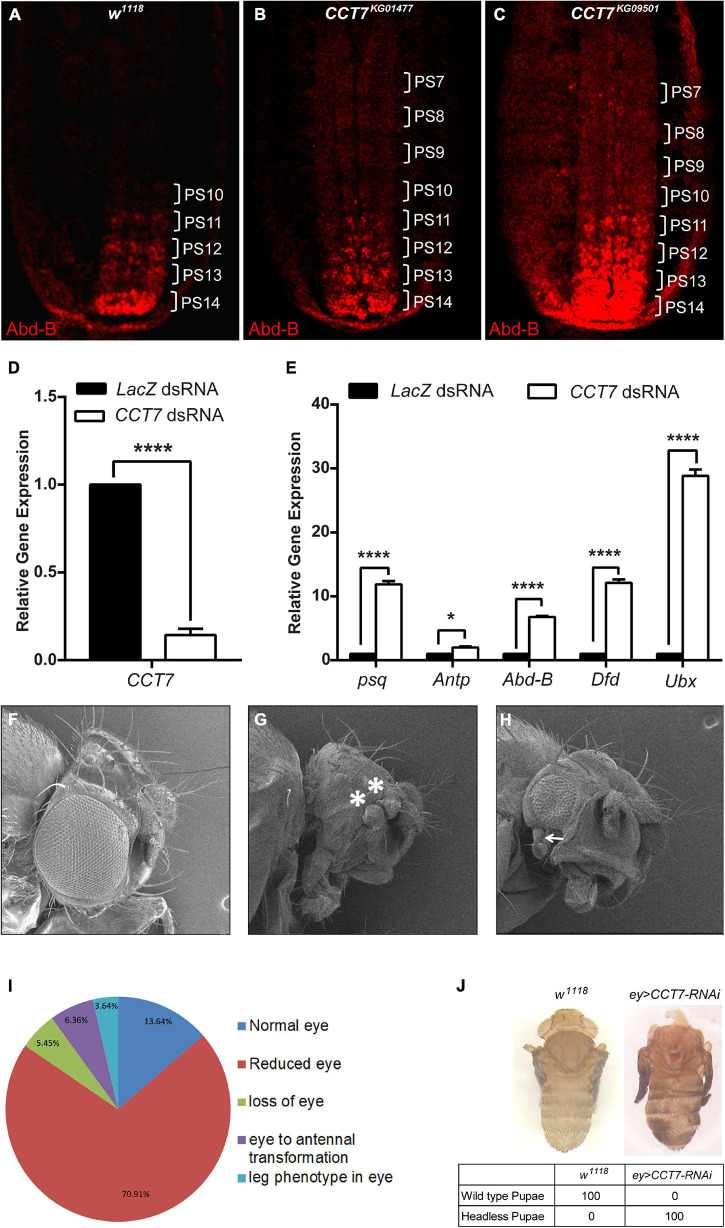
Depletion of chaperonin containing TCP-1 subunit 7 (CCT7) enhances the expression of Polycomb (Pc) group (PcG) target genes. **(A–C)** Enhanced levels of Abd-B expression observed in *CCT7* mutants compared with *w^1118^*. Stage 15 embryos of *w^1118^* **(A)** and homozygous mutant embryos for *CCT7*^*KG01477*^ **(B)** and *CCT7*^*KG09501*^ **(C)** were stained with Abd-B antibody. Homozygous *CCT7* embryos **(B,C)** showed increased Abd-B expression (parasegments marked as PS7-PS14) as compared to *w^1118^* embryos **(A)** used as control. All embryos are oriented with their anterior ends upward. **(D)** As compared to *LacZ* dsRNA-treated cells, knockdown of *CCT7* showed drastic reduction in mRNA levels of *CCT7* in D.Mel-2 cells. **(E)** Significantly increased levels of *psq*, *Antp*, *Abd-B*, *Dfd*, and *Ubx* expression determined in *CCT7* knockdown cells **(D)** using real-time PCR compared with *LacZ* dsRNA-treated cells. Error bars represent SEM for two independent experiments. Statistical significance was calculated using two-way ANOVA (* *p* ≤ 0.05, **** *p* ≤ 0.0001). **(F–H)** Eye-specific knockdown of *CCT7* shows a range of homeotic phenotypes, i.e., transformation of eye to duplicated antenna (marked with asterisks, **G)** and appearance of a leg-like appendage in addition to reduction in eye size (marked with arrow, **H**). **(I)** Pie chart representing the frequency of adult phenotypes resulting from eye-specific knockdown of *CCT7*. A total of 110 adult flies were observed under a stereo microscope (Nikon, C-DSS230) using white light and scored for aberrant eye phenotypes. Phenotypes of adult flies obtained as a result of knockdown of *CCT7* were categorized as follows: normal eye (13.64%), reduced eye (70.91%), loss of eye (5.45%), eye to antennal transformation (6.36%), and appearance of rudimentary leg in eye (3.64%). **(J)** Eye-specific knockdown of *CCT7* using a different and stronger RNAi line resulted in 100% lethality of the progeny at the pupal stage (right) as compared to *w^1118^* control (left). Headless pupal lethal pharate adult observed upon depletion of *CCT7* in the eye dissected out from the pupal case (right) as compared to *w^1118^* control (left).

Since a strong genetic interaction of *CCT7* with the PcG system was observed, we aimed to investigate if *CCT7* contributes to maintenance of repression by PcG. To this end, 12-h-old homozygous *CCT7* mutant embryos were stained with Abd-B antibody and compared with *w^1118^* embryos of the same age, which were used as control. In *w^1118^* embryos at this stage, *Abd-B* is expressed at progressively increasing level from parasegment 10 (PS10) to PS14, exhibiting strongest expression in PS14 (Celniker et al., 1989; Delorenzi and Bienz, 1990) (Figure 2A). As compared to *w^1118^* control, *CCT7* mutant embryos showed an increase in Abd-B expression in the anterior parasegments (Figures 2B,C). This increased Abd-B expression is a hallmark of PcG mutants (Simon et al., 1992). Additionally, *CCT7* was knocked down in D.Mel-2 cells using dsRNA (Figure 2D), and expression of PcG target genes was analyzed using quantitative real-time PCR. As compared to *LacZ* dsRNA-treated cells used as control, depletion of CCT7 resulted in a significant upregulation of *psq* (*pipsqueak*), *Antp* (*Antennapedia*), *Abd-B* (*Abdominal B*), *Dfd* (*Deformed*), and *Ubx* (*Ultrabithorax*) (Figure 2E), which are known PcG targets (Schuettengruber et al., 2009; Schwartz et al., 2010; Enderle et al., 2011). Next, *CCT7* was knocked down in flies using eye-specific GAL4 driver line (Figures 2F–H). Depletion of CCT7 resulted in transformation of eye to duplicated antenna (Figure 2G) and appearance of a leg-like appendage besides reduced eye size (Figure 2H). The adult flies obtained after eye-specific knockdown of *CCT7* were categorized as showing normal eye, reduced eye, loss of eye, eye to antennal transformation, and leg phenotype in the eye. The majority of flies, i.e., 70.91%, exhibited reduced eye size, 5.45% flies showed loss of eye phenotype, 6.36% flies presented eye to antennal transformation, 3.64% flies developed a rudimentary leg in the eye, and only 13.64% developed normal eye phenotype (Figure 2I). Additionally, depletion of CCT7 in the eye using a different RNAi line of CCT7 resulted in 100% pupal lethal phenotypes (Figure 2J). Interestingly, after eye-specific knockdown of CCT7, the F1 progeny never hatched and the late-stage pupae were dissected to observe the headless phenotype (Figure 2J, right) as compared to *w^1118^* control pupae (Figure 2J, left). The aberrant eye phenotypes and the headless pupal lethal pharate adults observed upon depletion of CCT7 may be attributed to the ectopic expression of *Antp* in eye–antennal imaginal discs (Gibson and Gehring, 1988; Plaza et al., 2001; Zhu et al., 2018). Similar pupal lethal phenotypes have been reported in eye-specific knockdown of all eight subunits of TRiC chaperonin complex (Kim and Choi, 2019). Together, these results suggest that PcG requires CCT7 to maintain repression.

### *Chaperonin Containing TCP-1 Subunit 7* Binds to Chromatin at Polycomb Group Targets

**FIGURE 3:**
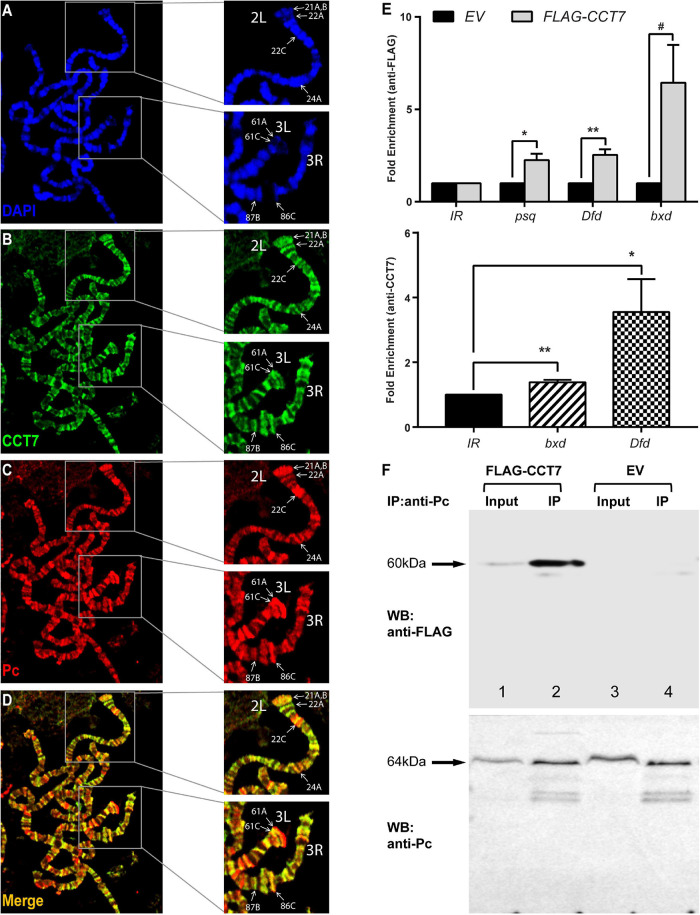
Chaperonin containing TCP-1 subunit 7 (CCT7) co-localizes and interacts with Polycomb (Pc). **(A–D)** Polytene chromosomes prepared from third instar larvae expressing Myc-CCT7 were stained with anti-Myc **(B)** and anti-Pc **(C)** antibodies. Myc-CCT7 was observed to co-localize with Pc at numerous loci, seen as yellow bands in merge **(D)**. In particular, co-localization of CCT7 with Pc at well-known Pc-binding sites in chromosome arms 2L, 3L, and 3R, on polytene chromosomes reported previously (Zink and Paro, 1989; Decamillis et al., 1992; Rastelli et al., 1993), is marked with white arrows. **(E)** Chromatin immunoprecipitation (ChIP) performed from FLAG-CCT7-expressing cells using anti-FLAG antibody showed CCT7 enriched at known Polycomb group (PcG) target genes, i.e., *psq*, *Dfd*, and *bxd* as compared to empty vector (*EV*) control cells (upper panel). Moreover, ChIP performed on 2–4-h-old *Drosophila* embryos using CCT7 antibody showed association of CCT7 at known Pc-binding sites in *bxd* and *Dfd* regions (lower panel). Intergenic region (*IR*) represents the region corresponding to chromosome arm 2R where Pc is normally not bound (Papp and Müller, 2006). ChIP DNA was quantified using real-time PCR, and data were normalized using fold enrichment over *IR*. Error bars represent SEM for three independent experiments. Statistical significance was calculated using *t* test (* p ≤ 0.05, ** p ≤ 0.01, # p = 0.056 trend toward significance). Schematic of the primers used in real-time PCR analysis and their locations are represented as blue lines in Supplementary Figure 2C. **(F)** Pc biochemically interacts with CCT7. Co-immunoprecipitation (Co-IP) was performed using anti-Pc antibody from *FLAG-CCT7*-expressing stable cells and *EV* control cells. Western blot with anti-FLAG (WB: anti-FLAG, upper) showed specific enrichment of FLAG-CCT7 in the IP sample (lane 2) of *FLAG-CCT7*-expressing cells as compared to control IP from *EV* cells (lane 4). Western blot with anti-Pc (WB: anti-Pc, lower) showed specific and equal enrichment of Pc in IP from both *FLAG-CCT7*-expressing cells and *EV* cells. Input represents 1% of total cell lysates from *FLAG-CCT7* (lane 1) and *EV* (lane 3) cells. The Ponceau S staining corresponding to the Western blot is shown in Supplementary Figure 2D.

Since homozygous mutation of *CCT7 in vivo* and its depletion in cells result in de-repression of PcG targets, it was questioned if CCT7 associates with chromatin at PcG targets and contributes to maintenance of repression. To address this question, transgenic flies expressing *Myc-CCT7* (Supplementary Figure 2A) under GAL4-inducible promoter were generated. Polytene chromosomes were prepared from third instar larvae expressing *Myc-CCT7* in salivary glands and stained with anti-Myc and anti-Pc antibodies (Figures 3A–D). It was observed that CCT7 co-localizes with Pc at multiple sites (Zink and Paro, 1989; Decamillis et al., 1992; Rastelli et al., 1993) on polytene chromosomes (Figure 3D). Next, ChIP was performed from *Drosophila* S2 stable cells expressing *FLAG-CCT7* using anti-FLAG antibody (Supplementary Figure 2B). ChIP from *EV* control cells expressing FLAG-epitope under copper-inducible promoter was used as a control. ChIP DNA was quantified by real-time PCR analysis using primers specific for known PcG-binding sites in *psq*, *Dfd*, and *bxd* loci (Schuettengruber et al., 2009; Schwartz et al., 2010; Enderle et al., 2011) (Supplementary Table 2). As compared to ChIP from *EV* control cells, FLAG-CCT7 was found to be enriched at *psq, Dfd*, and *bxd* (Figure 3E, upper panel). Importantly, CCT7 was not present at an intergenic region (*IR*) used as control where Pc does not bind (Figure 3E). Moreover, ChIP, using CCT7-specific antibody, performed from *Drosophila* embryos showed that CCT7 binds to known PcG-binding sites at *bxd* and *Dfd* (Figure 3E, lower panel). Based on the chromatin association of CCT7 at PcG target genes, it was assumed that CCT7 may biochemically interact with Pc and facilitate maintenance of silencing by PcG. To investigate molecular interaction between CCT7 and Pc, Co-IP was performed on stable cells containing *FLAG-CCT7* transgene. Total cell lysates from *EV* control and *FLAG-CCT7* stable cells were subjected to pulldown with anti-Pc antibody and subsequently analyzed on a Western blot that was probed with anti-FLAG and anti-Pc antibodies. As compared to Co-IP from *EV* control, immunoprecipitation with anti-Pc specifically resulted in enrichment of FLAG-CCT7 from stable cells expressing FLAG-CCT7 (Figure 3F), indicating that CCT7 interacts with Pc in *Drosophila* cells. Since CCT7 is found to associate with chromatin at PcG target genes (Schuettengruber et al., 2009; Schwartz et al., 2010; Enderle et al., 2011), as evidenced by ChIP and co-localization of CCT7 with Pc on polytene chromosomes, it suggests that this nuclear interaction between CCT7 and Pc maintains gene silencing.

### *Chaperonin Containing TCP-1 Subunit 7* Facilitates Nuclear Localization and Chromatin Association of Polycomb

**FIGURE 4:**
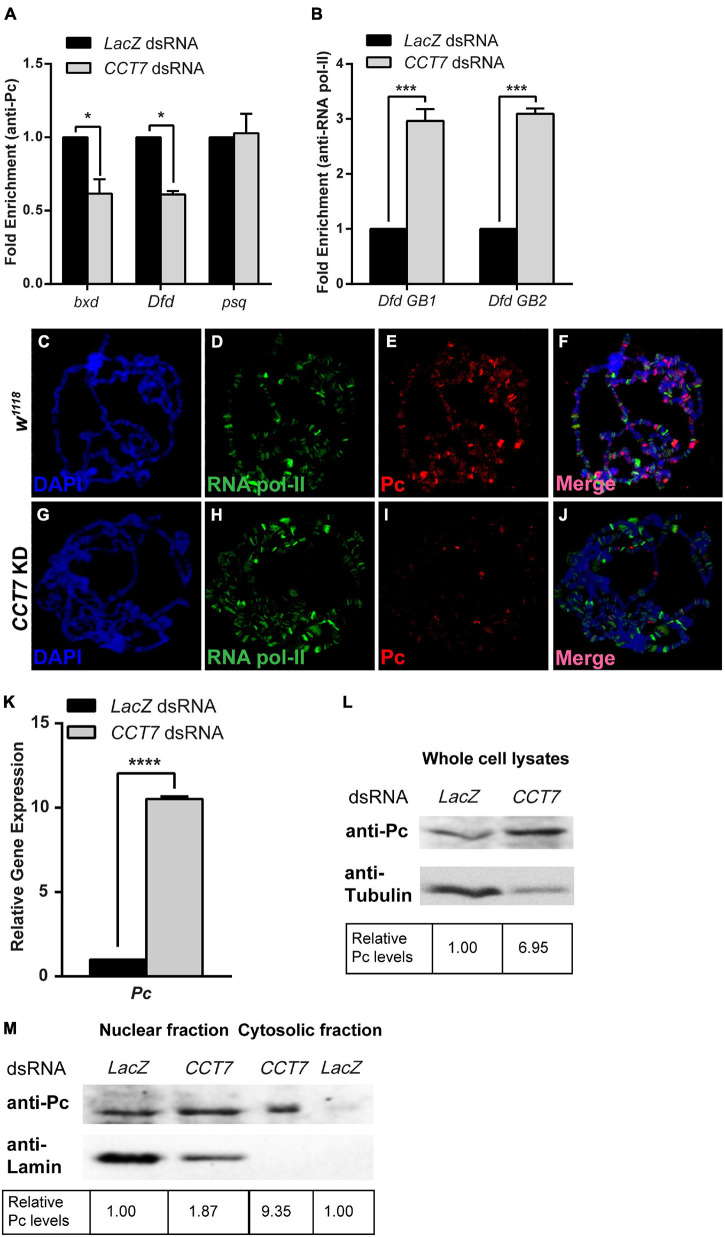
Chaperonin containing TCP-1 subunit 7 (CCT7) is required for association of Polycomb (Pc) at chromatin. **(A)** Chromatin immunoprecipitation (ChIP) using anti-Pc antibody from *CCT7* knockdown cells showed decreased enrichment of Pc at *bxd* and *Dfd* as compared to *LacZ* dsRNA-treated control cells. ChIP was analyzed using fold enrichment over input. Error bars represent SEM for two independent experiments. Statistical significance was calculated using two-way ANOVA (* *p* ≤ 0.01). **(B)** ChIP from cells treated with dsRNA against *CCT7* showed increased RNA pol-II along the gene body of *Dfd* (labeled *Dfd GB1* and *Dfd GB2*) as compared to control correlating with an increased expression of *Dfd* **(**Figure 2E) in CCT7-depleted cells. ChIP was analyzed using fold enrichment over input. Error bars represent SEM for two independent experiments. Statistical significance was calculated using two-way ANOVA (*** *p* ≤ 0.001). **(C–J)** Polytene chromosomes prepared from *w^1118^* and CCT7-depleted salivary glands and stained with anti-RNA pol-II and anti-Pc antibodies. As compared to RNA pol-II staining used as a positive control **(D,H)**, a strongly diminished binding of Pc was observed after depletion of CCT7 **(I)** when compared with *w^1118^* control **(E)**. **(K,L)** Drastic increase in expression of *Pc* in CCT7-depleted cells. CCT7-depleted cells displayed significantly higher expression of *Pc* in real-time PCR analysis **(K)** as well as on Western blot **(L)** when compared to control cells. Error bars represent SEM for two independent experiments. Statistical significance was calculated using *t*-test (**** *p* ≤ 0.0001). **(M)** Western blot probed with anti-Pc and anti-Lamin antibodies showing nuclear and cytosolic fractions isolated from CCT7-depleted and control cells. Besides its presence in the nucleus, Pc is visible as enriched in the cytosolic fraction of CCT7-depleted cells as compared to the cytosolic fraction of *LacZ* dsRNA-treated control. The same blot probed with anti-Lamin served as a control for purity of nuclear and cytosolic fractions. Western blots were quantified using ImageJ software, and data were normalized using *LacZ* dsRNA-treated cells as control.

The genetic and molecular interaction of CCT7 with Pc intrigued us to ask if depletion of CCT7 has an impact on association of Pc at the chromatin. To address this question, ChIP was performed using anti-Pc antibody from cells treated with dsRNA against *CCT7* followed by real-time PCR analysis. As compared to control cells treated with *LacZ* dsRNA, ChIP from CCT7-depleted cells revealed reduced association of Pc at *bxd* and *Dfd* chromatin-binding sites (Figure 4A). As compared to control cells, Pc binding at *psq*, a PcG target gene that is actively transcribed, remained unchanged in CCT7-depleted cells (Figure 4A), and it served as control supporting the notion that knockdown of *CCT7* results in diminishing Pc from silent PcG target genes. Moreover, ChIP analysis of RNA pol-II binding in *CCT7* knockdown cells revealed that RNA pol-II was enriched along the gene body of *Dfd* (Figure 4B) that correlates with increased expression of *Dfd* in these cells (Figure 2E). For an *in vivo* validation of decreased association of Pc at chromatin after CCT7 depletion in cells, *CCT7* was knocked down specifically in salivary glands of third instar larvae by crossing *CCT7* RNAi flies with salivary gland-specific GAL4 (*Sgs-GAL4*) driver line. Polytene chromosomes were prepared from third instar larvae, and immunostaining was performed with Pc and RNA pol-II antibodies (Figures 4C–J). Polytene chromosomes from *w^1118^* larvae of the same age stained with Pc and RNA pol-II antibodies were used as control (Figures 4C–F). As compared to control (Figure 4E), Pc staining was drastically reduced on polytene chromosomes from salivary glands where CCT7 was depleted (Figure 4I). However, no such effect on RNA pol-II association with polytene chromosomes was observed in CCT7-depleted salivary glands when compared with polytenes from *w^1118^* control (Figures 4D,H). This Pc-specific effect of CCT7 depletion led us to investigate possible underlying effects on the expression of *Pc*. To this end, we knocked down *CCT7* using dsRNA and analyzed the expression of *Pc*. Interestingly, quantitative real-time PCR analysis revealed that expression of *Pc* was significantly increased in CCT7-depleted cells as compared to *LacZ* dsRNA-treated cells used as control (Figure 4K). This increase in the mRNA levels of *Pc* also correlates with increased Pc protein in *CCT7* knockdown cells as compared to control cells (Figure 4L). The increased expression of *Pc* in CCT7-depleted cells together with loss of Pc association with chromatin in the absence of CCT7 illustrates the importance of CCT7 in regulating Pc at both transcriptional and posttranslational levels. However, the increased expression of Pc in cells is contrary to the decreased association of Pc on chromatin when CCT7 was depleted (Figures 4A,I), which led us to investigate if CCT7 is required for proper localization of Pc within the cells. To address this question, we isolated nuclear and cytosolic fractions of CCT7-depleted cells and analyzed the fractions on a Western blot with anti-Pc antibody. Intriguingly, an increased amount of Pc was observed in the cytosolic fraction of *CCT7* knockdown cells when compared with control cells. However, amount of Pc in the nuclear fractions of CCT7-depleted and control cells remained the same. Absence of Lamin in the cytosolic fraction served as control and illustrates that there was no nuclear contamination in the cytosolic fraction. Therefore, it validates that the Pc observed in the cytosolic fraction is a specific consequence of CCT7 depletion (Figure 4M). Altogether, our data demonstrate that CCT7 facilitates gene silencing by regulating Pc at multiple levels. In particular, CCT7 helps in nuclear localization and chromatin association of Pc besides contributing to maintenance of gene expression of Pc gene. The increased expression of Pc in CCT7-depleted cells does not get associated with chromatin, suggesting that CCT7 is required for recruitment of Pc at chromatin. Importantly, the presence of an ample amount of Pc in cytoplasm in the absence of CCT7 suggests that CCT7 is more likely playing a role in chaperoning Pc to its chromatin-binding sites. Taken together, these results highlight a critical role for CCT7 in the maintenance of gene repression by Pc in the process of cell fate maintenance and epigenetic cell memory in *Drosophila*.

## Discussion

In order to understand better the complex relationship between genotype and phenotype, it is necessary to study the link between epigenetic pathways, cellular signaling, and environmental factors during development. Molecular chaperones represent a class of proteins, which rapidly respond to intracellular or extracellular environmental cues to initiate epigenetically heritable changes (Sawarkar and Paro, 2013; Condelli et al., 2019). Our results demonstrate a previously unknown genetic and molecular link between CCT7 and Pc, which is essential for maintaining heritable gene repression. We provide evidence that depletion of CCT7 results in reactivation of PcG target genes and leads to severe morphological defects. Since Pc dissociates from chromatin and is retained in cytoplasm as a consequence of diminished CCT7, we propose that CCT7 is required for chaperoning Pc to its targets on chromatin and maintenance of repression. Involvement of CCT7 in maintenance of silencing by Pc has revealed a novel epigenetic modulator of Pc that ensures robustness of gene expression during development. Such an interaction of molecular chaperone Hsp90 with epigenetic factors like TRX has previously been reported in *Drosophila* (Sollars et al., 2003; Tariq et al., 2009; Sawarkar et al., 2012). In contrast to the role of Hsp90 in gene activation by trxG, little is known about the role of molecular chaperones in PcG-mediated gene regulation and the effects that they may have on epigenetic cell memory.

Genetic evidence presented here demonstrates that TRiC members, *CCT7* and *CCT5*, exhibit PcG-like behavior by enhancing extra sex comb phenotype of *Pc*. Appearance of extra sex combs on the second and third pairs of legs in *Drosophila* males is a classical mutant phenotype in *Pc* heterozygotes (Riley et al., 1987; Pattatucci and Kaufman, 1991). Strong enhancement of extra sex comb phenotype by two different alleles of *CCT7* and *CCT5* suggests that TRiC subunits are possibly required for the proper function of one or more PcG proteins. Such an enhancement of extra sex comb phenotype has also been described for molecular chaperone *Hsc4* mutant in *Drosophila* (Mollaaghababa et al., 2001; Wang and Brock, 2003). The PcG-like behavior of *CCT7* mutants was supported by the antagonistic effect of *CCT7* in *CCT7/trx^1^* double mutants where it suppresses the *trx^1^* mutant (Ingham and Whittle, 1980) phenotype. These genetic data are substantiated by an increased expression of homeotic and non-homeotic targets of PcG, which is explained by dissociation of Pc from chromatin when CCT7 was depleted, thus reinforcing the Pc-specific effect of *CCT7* in transcriptional cellular memory.

Based on the genetic and molecular evidence presented here, it is plausible to assume that CCT7 may facilitate the recruitment of Pc on chromatin. This hypothesis explains the strong enhancement of extra sex comb phenotype and reactivation of PcG targets, since mutations in *CCT7* chaperonin might have reduced the chromatin-associated Pc protein to maintain gene repression. The mechanistic link between Pc and CCT7 may be similar to the role played by TRiC in priming histone deacetylase (HDAC)3 for its nuclear localization and potential interaction with SMRT to form active SMRT-HDAC3 repression complex (Guenther et al., 2002). Moreover, TRiC interacts with HDAC1/HDAC2 (Banks et al., 2018) and SWI/SNF chromatin remodeling complexes (Banks et al., 2018) and acts as a checkpoint in the assembly of basal transcription factor TFIID (Antonova et al., 2018), highlighting the importance of TRiC at different nodes in regulating gene expression states. Molecular interactions of TRiC with HDACs and SWI/SNF complexes and its role in holo TFIID assembly indicate that TRiC chaperonins may have a dual role in silencing and activation of gene expression. Such a dual role of molecular chaperones in transcriptional activation (Sollars et al., 2003; Tariq et al., 2009; Sawarkar et al., 2012) and repression (Laskar et al., 2011; Okazaki et al., 2018; Sun et al., 2020) has also been reported for Hsp90.

Despite an increased amount of Pc in CCT7-depleted cells, there was decreased association of Pc at chromatin. This may be due to a large amount of Pc retained in the cytosol in the absence of CCT7. It would be interesting to determine the composition of Pc-interacting proteins in wild-type cells and in cells deficient for CCT7. If CCT7 is required for assembly of Pc-containing protein complexes, different Pc-interacting proteins will be purified in the absence of CCT7. Since it is possible to assemble functional PRC1 core complex *in vitro* (Francis et al., 2001) in the absence of CCT7, it suggests that CCT7 may have a specific effect on association of Pc at chromatin. The depletion of Pc, but not RNA pol-II from chromatin, in the absence of CCT7 further suggests that CCT7 has a specific role in Pc-mediated repression of key developmental genes. The increased expression of PcG targets in CCT7-depleted cells as a result of decreased association of Pc and subsequent release of RNA pol-II from the paused state is in accordance with the previously reported function of Pc in holding RNA pol-II in its paused state (Lis, 1998; Breiling et al., 2001; Dellino et al., 2004) on silent genes. While we provide evidence that Pc requires CCT7 to maintain repression, an indirect effect of CCT7 on association of Pc at chromatin through one of its client proteins cannot be ruled out.

The genetic and molecular interplay of TRiC at a multitude of cellular signaling pathways together with evolutionarily conserved PcG suggests that it may serve as a major hub similar to Hsp90 to maintain cellular homeostasis. The molecular interaction between CCT7 and Pc illustrates how gene expression states can rapidly be modulated that eventually may lead to accumulation of epigenetic variation and possible phenotypic variation. Further molecular and biochemical characterization of CCT7–Pc nexus is required to reveal the mechanistic details about how this intricate relationship contributes to the complex interplay between genotype and phenotype.

## Materials and Methods

### Fly Strains

The following fly strains were obtained from Bloomington *Drosophila* Stock Center (BDSC): *CCT7*^*KG01477*^ (BDSC: 13446), *CCT7*^*KG09501*^ (BDSC: 15191), *Pc^1^* (*Pc^1^/TM3Ser*) (gift from Renato Paro), *Pc*^*XL5*^ (*Pc*^*XL5*^*/TM3Ser, Sb*) (gift from Renato Paro), *trx^1^* (*trx*^1^*/TM1*) (BDSC: 2114), *CCT5^06444^* (BDSC: 12315), and *CCT5*^*K06005*^ (BDSC: 10393). The fly strains obtained from Vienna *Drosophila* Resource Center include *CCT7*^*KK101540*^ (VDRC: v108585) and *CCT7*^*GD13899*^ (VDRC: v28895). Moreover, fly strains used for polytene staining of transgenic flies include *P{w*^+*mC*^*UASp-CCT7-MYC}* [generated in-house by injecting *w^1118^* (BDSC: 5905) embryos with *P{w*^+*mC*^*UASp-CCT7-MYC}* construct, and transgenic flies were selected using mini-white gene as selection marker] and *pTub-GAL4/Tb* [BDSC: 5138 (*{tubP-GAL4}LL7/TM3, Sb[1] Ser[1]*) balanced with *Tb* balancer]. Additionally, fly strains used for immunostaining of polytene chromosomes after RNAi include *CCT7*^*GD13899*^ (VDRC: v28895) and *P{Sgs3-GAL4.PD}* (BDSC: 6870).

The fly strains used to perform eye-specific knockdown were *CCT7*^*KK101540*^ (VDRC: v108585), *CCT7*^*GD13899*^ (VDRC: v28895), and *ey-GAL4* (gift from Renato Paro). For immunostaining of homozygous mutant embryos, *CCT7*^*KG01477*^ and *CCT7*^*KG09501*^ were balanced with a green fluorescent protein (GFP) balancer chromosome.

### Antibodies

The antibodies and their dilutions used in this study are as follows: mouse anti-Abd-B [Developmental Studies Hybridoma Bank (DSHB), 1A2E9] [immunofluorescence (IF) 1:40], rabbit anti-Pc [IF 1:20, Western blotting (WB) 1:4,000] (gift from S. Hirose), mouse anti-Myc (Santa Cruz, 9E10) (IF 1:50, WB 1:1,000), mouse anti-FLAG M2 (Sigma Aldrich, F1804) (ChIP 5 μl, WB 1:1,000), mouse anti-Tubulin (Abcam, ab7291) (WB 1:2,000), mouse anti-RNA pol-II (Abcam, ab5408) (ChIP 2 μl, IF 1:100), mouse anti-Lamin (DSHB, ADL67.10-s) (WB 1:1,000), rabbit anti-CCT7 (Atlas antibodies, HPA008425) (ChIP 8 μl). Secondary antibodies used for WB were goat anti-rabbit immunoglobulin G (IgG) H&L [horseradish peroxidase (HRP)] (Abcam, ab6721) and goat anti-mouse IgG H&L (HRP) (Abcam, ab6789) at 1:5,000 dilution. Secondary antibodies for IF were Cy3-conjugated goat anti-rabbit IgG (H+L) (Thermo Fisher Scientific, A10520) and Alexa Fluor 488-conjugated goat anti-mouse IgG (H+L) (Thermo Fisher Scientific, A28175) at 1:100 dilution.

### Genetic Analysis

Two different alleles each of *CCT7* and *CCT5* were independently crossed with two different *Pc* alleles (*Pc^1^* and *Pc*^*XL5*^) at 25°C, while *w^1118^* flies crossed to *Pc* alleles were used as control. Males in the progeny of these crosses were scored for extra sex comb phenotype as described previously (Tariq et al., 2009). Similarly, both mutants of *CCT7* and *w^1118^* were crossed to *trx* mutant allele (*trx^1^*), and males in the progeny of these crosses were analyzed and scored for abdominal pigmentation phenotype as described previously (Ingham and Whittle, 1980; Umer et al., 2019).

### Immunohistochemistry

Transgenic flies carrying *P{w*^+*mC*^*UASp-CCT7-Myc}* were generated using FemtoJet Microinjector (Eppendorf) following standard protocol (Bienz et al., 1988). These transgenic flies were crossed with *pTub-GAL4/Tb* driver line to induce the expression of *CCT7-Myc*. Salivary glands from *non-Tb* third instar larvae expressing *CCT7-Myc* were isolated, and polytene chromosomes were stained with anti-Myc and anti-Pc antibodies as described previously (Zink and Paro, 1989). Briefly, third instar larvae were dissected to isolate salivary glands. Two pairs of salivary glands were incubated in a drop of 3.7% paraformaldehyde fixation solution for 15 min on a poly-L-lysine-coated slide. After 15 min, the salivary glands were squashed by placing a coverslip on top, and nuclei were broken apart with the help of a sharpened pencil. The slides were splash frozen in liquid nitrogen, and coverslip was snapped off in a single attempt with a razor blade. The slides were then washed twice in 1 × phosphate buffered saline (PBS) (137 mM NaCl, 2.7 mM KCl, 10 mM Na_2_HPO_4_, and 1.8 mM KH_2_PO_4_ at pH 7.4) and once in 1 × PBS + 1% Triton X-100 (PBST) for 10 min each. These polytene chromosomes were incubated in blocking buffer (5% non-fat milk in 1 × PBS) in a slide jar for 1 h at room temperature with vigorous shaking. After blocking, slides were rinsed in 1 × PBS solution and incubated with 30 μl of desired primary antibodies in a humid chamber overnight at 4°C. Next day, coverslips were removed by holding the slides in 1 × PBS, and slides containing polytenes were washed in blocking solution for 30–40 min at room temperature. These slides were incubated with appropriate secondary antibodies in the dark at room temperature. After 2 h of incubation with secondary antibodies, the slides were washed sequentially with wash buffer 1 (300 mM NaCl, 0.2% NP-40, 0.2% Tween20 in 1 × PBS) first and then with wash buffer 2 (400 mM NaCl, 0.2% NP-40, 0.2% Tween20 in 1 × PBS) for 10 min each in aluminum foil-covered slide jar with vigorous shaking. The slides were incubated with 4′,6-diamidino-2-phenylindole (DAPI) (250 ng/slide) (Sigma Aldrich, D9542) for 20–30 min in the dark. The coverslips were removed to mount the slides in Fluoromount-G Mounting Medium (Thermo Fisher Scientific, 00-4958). All images for polytene chromosomes were observed at ×60 optical zoom, and images were acquired using Nikon C2 Confocal Microscope. Images were acquired using the same laser settings for the corresponding experimental and control samples in a sequential manner.

For embryonic staining, stage 15 embryos were dechorionated in 3% sodium hypochlorite solution for 5 min, and GFP-negative embryos (homozygous for the mutations) were separated under an epifluorescent stereo microscope (Nikon, C-DSS230) followed by immunostaining with Abd-B antibodies following previously described protocol (Mitchison and Sedat, 1983). Briefly, the dechorionated embryos were transferred to 5-ml heptane solution in a vial, and an equal volume of 3.7% formaldehyde solution [in PEM (0.1 M PIPES, 1 mM EGTA, 1 mM MgCl_2_) buffer, pH 6.9 adjusted with KOH] was added, followed by vigorous shaking and incubation at room temperature. After 20 min, the lower formaldehyde layer was removed with the help of a pipette, and 5 ml methanol was added. After shaking vigorously for 15 s, the upper heptane layer was removed along with the embryos that were suspended at the interphase of the two layers. The two-third volume of vial was filled up with methanol and stored overnight at 4°C. On the following day, the embryos were transferred to a 1.5-ml tube and washed twice on an orbital rotor with 1 × PBS to remove methanol. Embryos were permeabilized and incubated in blocking buffer [0.3% Triton X-100 and 0.5% bovine serum albumin (BSA) in 1 × PBS] for 1 h on an orbital rotor at room temperature. The embryos were then incubated with appropriate primary antibodies overnight at 4°C on an orbital shaker. Next day, primary antibodies were removed with the help of pipette, and embryos were washed for 5 min with 1 × PBS thrice on a rotor followed by incubation with appropriate secondary antibodies for 2 h at room temperature. Finally, embryos were washed three times with 1 × PBS and incubated with DAPI solution for 30 min, followed by two washings with 1 × PBS and mounting of embryos on a microscope slide in Fluoromount-G Mounting Medium (Thermo Fisher Scientific, 00-4958). All incubations on the orbital shaker were performed at 30 rpm. All images were acquired using the Nikon C2 Confocal Microscope. All embryos, *w^1118^* control and homozygous *CCT7* mutants, were exposed to the same laser settings in a sequential manner.

### Microscopy

For analyzing loss of abdominal pigmentation, male flies of the desired genotypes were transferred to 70% ethanol to dehydrate. The dehydrated flies were then dissected under Olympus SZ51 stereomicroscope on a dissection slide with the help of fine needle. The abdominal portion of the flies were isolated and rehydrated in water for 5 min. After rehydration, abdomens were dissected for cuticle preparation. A coverslip was placed over the cuticle and observed under Nikon C-DSS230 epifluorescent stereomicroscope at ×3.5 magnification using white light for imaging. For analyzing the immunostaining data, embryos were observed at ×20 using Nikon C2 Confocal Microscope. Images were acquired by exposure of embryos to lasers, i.e., DAPI (excitation 405 nm and emission 447 nm) and Cy3 (excitation 561 nm and emission 785 nm) in sequential manner. All polytene chromosomes were visualized at ×60 magnification using Nikon C2 Confocal Microscope. Images were acquired by exposing to DAPI (excitation 405 nm and emission 447 nm), Alexa 488 (excitation 488 nm and emission 525 nm), and Cy3 (excitation 561 nm and emission 785 nm) in a sequential manner. Nikon NIS Elements image acquisition software (Version: 5.21.00) was utilized for imaging and analysis.

### dsRNA Synthesis

Specific regions used for *CCT7* knockdown in cells were selected from *Drosophila* RNAi Screening Center (DRSC), and their primers are detailed in Supplementary Table 2. Templates for the preparation of dsRNA were amplified by PCR from cDNA using T7-tailed oligonucleotides as primers. These templates were then used for *in vitro* transcription to synthesize dsRNA by using T7 Megascript kit following the manufacturer’s instructions (Thermo Fisher Scientific, AM1333).

### *Drosophila* Cell Culture

*Drosophila* S2 cells were cultured in Schneider’s *Drosophila* medium (Gibco, Thermo Fisher Scientific, 11720-034), supplemented with 10% fetal bovine serum (Gibco, Thermo Fisher Scientific, 10082147) and 1% penicillin-streptomycin (Gibco, Thermo Fisher Scientific, 15140122) at 25°C. *Drosophila* S2 cells adjusted to serum-free growth medium were cultured in Express Five SFM (Gibco, Thermo Fisher Scientific, 10486025) supplemented with 20 mM GlutaMAX (Gibco, Thermo Fisher Scientific, 35050061) and 1% penicillin-streptomycin (Gibco, Thermo Fisher Scientific, 15140122).

### Real-Time Polymerase Chain Reaction Analysis

The expression of PcG target genes was analyzed by performing real-time PCR. Briefly, cells were harvested, and total RNA was extracted using TRIzol reagent following the manufacturer’s protocol (Thermo Fisher Scientific, 15596026). Total RNA was subjected to DNase (TURBO DNA-free Kit, Thermo Fisher Scientific, AM1907) treatment to get rid of any contaminating genomic DNA. The DNase-treated RNA was then used to make cDNA using SuperScript III First-Strand Synthesis System following the manufacturer’s protocol (Thermo Fisher Scientific, 18080051). Gene expression was quantified by real-time PCR (Applied Biosystems 7500) using 50 ng of cDNA for each reaction employing Power SYBR Green PCR Master Mix (Thermo Fisher Scientific, 4367659). The relative gene expression was calculated as fold enrichment using ΔΔCt method (Green and Sambrook, 2018).

### Western Blotting

Cells were lysed in ice-cold lysis buffer (150 mM NaCl, 0.05 M Tris, 1% Triton X-100) supplemented with the following protease inhibitors: pepstatin (0.5 μg/ml), leupeptin (0.5 μg/ml), aprotinin (0.5 μg/ml), and phenylmethylsulfonyl fluoride (PMSF) (1 mM). After centrifugation at 10,000 rpm, supernatant was collected in fresh tubes, mixed with 2 × reducing sample buffer, and boiled at 95°C for 5 min. The proteins were resolved on 10% sodium dodecyl sulphate-polyacrylamide gel electrophoresis (SDS–PAGE) and transferred onto nitrocellulose membranes that were blocked with 5% milk for 30 min before probing with the appropriate antibodies overnight at 4°C. Secondary antibodies, HRP-conjugated, were used at 1:10,000 dilution, and blots were developed using enhanced chemiluminescence (ECL) reagents (GE Healthcare, RPN 2108). The experiments were reproduced thrice.

### Generation of Stable Cell Line and Transgenic Flies

To generate vectors expressing tagged proteins, RNA extracted from *Drosophila* S2 cells was used to synthesize cDNA. Primers designed for Gateway Cloning were used to amplify *CCT7* CDS from cDNA that was then cloned into *pENTR/D-TOPO* vector (Thermo Fisher Scientific, K240020). LR Clonase reactions (Thermo Fisher Scientific, 11791020) were set up with *Drosophila* Genomics Resource Center (DGRC) vectors for both cell culture and fly transformation containing either Myc or FLAG tags to prepare epitope-tagged CCT7. Primer details can be found in Supplementary Table 2.

For the generation of stable cell lines, *pMT-FLAG-CCT7* plasmid was transfected into *Drosophila* S2 cells using Effectene transfection reagent (Qiagen, 301425). Transfected cells were selected with Hygromycin B (Roche, 10843555001) to a final concentration of 250 μg/ml. Finally, cells were induced with 500 μM CuSO_4_ for 72 h, and stable cells were confirmed for the expression of FLAG-CCT7 by WB with anti-FLAG antibody.

Fly transformation vector expressing Myc-CCT7, under the control of *UASp*, was used to generate transgenic fly lines by injecting *w^1118^* embryos using standard protocol (Bienz et al., 1988). Transgenic flies were confirmed by WB with anti-Myc antibody.

### Chromatin Immunoprecipitation

ChIP was performed as described previously (Tariq et al., 2009) with slight modifications. Briefly, 3 × 10^7^ cells were fixed at room temperature with 1% formaldehyde for 10 min. Cross-linking was stopped by the addition of glycine to a final concentration of 0.125 M. Cells were washed with 1 × PBS and lysed with Buffer A (10 mM Tris pH 8.0, 0.25% Triton X-100, 10 mM EDTA, 0.5 mM EGTA) followed by two washes with Buffer B (10 mM Tris pH 8.0, 200 mM NaCl, 1 mM EDTA, 0.5 mM EGTA). Cells were sonicated in 300 μl of sonication buffer (10 mM Tris pH 8.0, 1 mM EDTA, 0.5 mM EGTA) using Bioruptor (Diagenode) at high setting for 15–25 min (30 s ON, 30 s OFF) such that chromatin fragment sizes were between 100 and 500 bp. Sonicated chromatin was centrifuged at 13,000 rpm for 10 min at 4°C, and the cleared chromatin was stored at −80°C. Chromatin was diluted with 2 × radioimmunoprecipitation assay (RIPA) buffer (20 mM Tris pH 8.0, 2 mM EDTA, 280 mM NaCl, 2%Triton X-100, 0.2% SDS, 0.2% sodium deoxycholate) and precleared by incubating with DYNA beads (Invitrogen) for 2 h at 4°C with 20 rpm rotation. Precleared chromatin was incubated with the appropriate antibody overnight at 4°C on an orbital shaker with 20 rpm. Immunocomplexes were pulled down with DYNA beads. The beads were washed five times with 1 × RIPA, once with LiCl buffer (10 mM Tris pH 8.0, 250 mM LiCl, 1 mM EDTA, 0.5% NP-40, 0.5% sodium deoxycholate) and twice with 1 × TE (10 mM Tris pH 8.0, 1 mM EDTA). Chromatin was eluted by incubating beads at 65°C with 500 μl of freshly made elution buffer (0.1 M sodium bicarbonate, 1% SDS) for 15 min. Reverse cross-linking of chromatin was carried out overnight with NaCl at 65°C followed by Proteinase K (Thermo Fisher Scientific, 25530049) treatment for 2 h. Reverse cross-linked chromatin was extracted using phenol-chloroform followed by ethanol precipitation. All buffers were supplemented with PMSF (Sigma Aldrich, P7626), aprotinin (Sigma Aldrich, 9087-70-1), leupeptin (Thermo Fisher Scientific, AAJ18413LB0), and pepstatin (Thermo Fisher Scientific, 20037) protease inhibitors.

ChIP was performed from stable cell lines expressing FLAG-CCT7 or empty vector (EV) induced with 500 μM CuSO_4_ for 72 h. Purified ChIP DNA from each reaction was quantified using quantitative real-time PCR (Applied Biosystems Inc.). Chromatin enrichment was calculated as fold enrichment over input using ΔΔCt method (Tariq et al., 2003). For ChIP after knockdown of *CCT7*, cells were treated with 10 μg/ml of dsRNA for 5 days. Knockdown was confirmed using real-time PCR (Applied Biosystems Inc.). ChIP was then performed with 1 × 10^7^ cells with appropriate antibodies, and ChIP DNA was quantified using real-time PCR (Applied Biosystems Inc.). Chromatin enrichment, for ChIP after knockdown of *CCT7*, was calculated as fold enrichment over input using ΔΔCt method as described previously (Tariq et al., 2003). All the ChIP experiments were performed in duplicate. Primers used in ChIP analysis can be found in Supplementary Table 2. Primer locations are depicted in Supplementary Figure 2C.

For ChIP from embryos, 2–4-h-old embryos were sonicated as described previously (Bonnet et al., 2019). Briefly, embryos were washed and dechorionated using sodium hypochlorite for 40–60 s. The dechorionated embryos were washed extensively with water and 1 × PBST (0.1% Triton X-100 in 1 × PBS) and cross-linked for 15 min with 1% formaldehyde solution (made in heptane). Next, 0.125 M glycine was used to stop the cross-linking. Fixed embryos were washed twice with 1 × PBST for 10 min each. After washing, the embryos were transferred to a 1.5-ml tube and flash-frozen in liquid N2 followed by storage at −80°C. Next day, the frozen embryos were placed in ice to thaw and resuspended in 1 ml of ice-cold 1 × PBST supplemented with protease inhibitors, i.e., pepstatin (0.5 μg/ml) (Thermo Fisher Scientific, 20037), leupeptin (0.5 μg/ml) (Thermo Fisher Scientific, AAJ18413LB0), aprotinin (0.5 μg/ml) (Sigma Aldrich, 9087-70-1), and PMSF (1 mM) (Sigma Aldrich, P7626). Embryos were homogenized using Dounce homogenizer (KIMBLE KONTES Dounce Tissue Grinder, 885300-0015) and transferred into 15-ml tube. After centrifugation at 400 g for 1 min at 4°C, the debris was removed. The supernatant was transferred into a fresh 15-ml tube and centrifuged at 1,100g for 10 min to pellet down the nuclei. The supernatant was discarded, and pellet was resuspended in ice-cold lysis buffer supplemented with protease inhibitors described above. The cells were homogenized using Dounce homogenizer on ice by applying 20 strokes. The homogenate was centrifuged at 2,000g for 4 min at 4°C, and nuclear pellet was resuspended in 1 ml ice-cold nuclear lysis buffer (10 mM EDTA, 0.5% N-lauroylsarcosine, 50 mM HEPES, pH 8.0) containing protease inhibitors and incubated at room temperature for 20 min. After incubation, 1 ml of ice-cold nuclear lysis buffer was added and aliquoted into 1.5-ml microfuge tubes. The chromatin was subjected to sonication for 22 cycles (30 s ON/30 s OFF) using Bioruptor (Diagenode). The sonicated chromatin was centrifuged at 14,000 rpm for 10 min at 4°C, and supernatant was collected for performing ChIP as described above with appropriate antibodies.

### Co-immunoprecipitation

Co-immunoprecipitation (Co-IP) was performed on *FLAG-CCT7*-expressing stable cells, and *EV* cells induced for 72 h were lysed in lysis buffer (50 mM NaCl, 50 mM Tris pH 8.0, 1 mM EDTA, 0.2 mM Na_3_VO_4_, 1% Triton X-100) supplemented with the following protease inhibitors: pepstatin (0.5 μg/ml) (Thermo Fisher Scientific, 20037), leupeptin (0.5 μg/ml) (Thermo Fisher Scientific, AAJ18413LB0), aprotinin (0.5 μg/ml) (Sigma Aldrich, 9087-70-1), and PMSF (1 mM) (Sigma Aldrich, P7626). Lysis was carried out on ice for 10 min followed by sonication (three cycles each of 5 s ON and 10 s OFF) to shear the DNA. The lysate was centrifuged at 14,000 rpm for 10 min, and supernatant was transferred to a fresh tube. The samples were quantified by Bradford reagent (Thermo Fisher Scientific), and equal proteins were taken as immunoprecipitated (IP) samples from both *FLAG-CCT7* and *EV* cells. Here, 1% of the total lysates were used as input samples. The lysates were then incubated with anti-Pc antibody at 4°C with 20 rpm rotation on an orbital shaker. After 2 h of incubation, the immune complexes were incubated with DYNA beads (Invitrogen) for 4 h at 4°C with 20 rpm rotation. The beads were then washed thrice with lysis buffer for 5 min each to remove the unbound proteins. Finally, the IP samples together with their respective input samples were resuspended in 2× SDS loading dye, boiled at 95°C for 5 min, and analyzed on a Western blot with respective antibodies. The experiment was performed thrice, and the results were consistent.

### Analysis of Nuclear and Cytosolic Fractions

Cells were harvested and centrifuged at 10,000 rpm for 5 min at 4°C followed by lysis in Buffer A (pH 7.9, 10 mM HEPES, 1 mM EDTA, 1 mM EGTA, 100 mM KCl, 1 mM DTT, 0.5% NP-40 supplemented with protease and phosphatase inhibitors) for 15 min on ice. After mixing gently on a vortex for 30 s, it was centrifuged at 10,000 rpm for 15 min. The supernatant was collected in a fresh tube as cytosolic fraction, and the pellet was resuspended in 2× SDS loading buffer and labeled as nuclear fraction. Nuclear and cytosolic fractions isolated from both CCT7-depleted and control cells were analyzed on Western blot with appropriate antibodies, and the experiment was performed thrice.

## Data Availability Statement

The original contributions presented in the study are included in the article/Supplementary Material, further inquiries can be directed to the corresponding author.

## Author Contributions

NS, JA, ZU, and MT designed the research and wrote the manuscript. NS, JA, MK, MB, and MS performed the research. NS, JA, AF, and MT analyzed the data. All authors contributed to the article and approved the submitted version.

## Conflict of Interest

The authors declare that the research was conducted in the absence of any commercial or financial relationships that could be construed as a potential conflict of interest.

## Publisher’s Note

All claims expressed in this article are solely those of the authors and do not necessarily represent those of their affiliated organizations, or those of the publisher, the editors and the reviewers. Any product that may be evaluated in this article, or claim that may be made by its manufacturer, is not guaranteed or endorsed by the publisher.

## Supplementary Material

Supplementary Figure 1 *CCT7* and *CCT5* mutants enhance extra sex comb phenotype of *Pc*. **(A–D)** *CCT7*^*KG09501*^ and *CCT5*^*K06005*^ mutants were crossed to two different *Pc* (*Pc^1^* and *Pc*^*XL5*^) alleles and double mutant (*CCT7/Pc and CCT5;Pc*) male flies in the progeny were scored for extra sex comb phenotype. Heterozygous male flies for *Pc* (*+/Pc*) from the cross of *w^1118^* with *Pc* alleles were used as control. *CCT7*^*KG09501*^ mutation enhanced the extra sex comb phenotype of both *Pc^1^* and *Pc*^*XL5*^ in double mutant *CCT7*^*KG09501*^*/Pc^1^* **(A)** and *CCT7*^*KG09501*^/Pc^*XL5*^ **(B)** as compared to control. Similarly, *CCT5*^*K06005*^ mutant showed increase in the extra sex comb phenotype in double mutant *CCT5*^*K06005*^*; Pc^1^* **(C)** and *CCT5*^*K06005*^*;Pc*^*XL5*^ **(D)** progeny as compared to control. Severity of phenotype and statistical analysis was performed as described in Figure 1.

Supplementary Figure 2 Western blot confirms the expression of epitope-tagged *CCT7* transgene. **(A)** Western blot showed Myc-tagged-CCT7 specifically detected in larval extracts where the expression of *UAS-Myc-CCT7* was induced by crossing with *pTub-GAL4* (+) driver line as compared to control (−). **(B)** Western blot showed the expression of FLAG-CCT7 in stable cells induced with 500μM CuSO_4_ (+) as compared to un-induced control (-). Tubulin was used as loading control. **(C)** Schematic of the DNA regions amplified using specific primers, represented as blue lines, for real time PCR analysis of ChIP DNA. **(D)** Ponceau stained blot corresponding to the Co-IP performed from *Drosophila* cells (Figure 3F).

Supplementary Table 1 Percentages of extra sex comb flies scored in different categories corresponding to Figures 1A–D and Supplementary Figures 1A–D.

Supplementary Table 2 List of primers used in this study.

## References

[B1] Anderson M. Fair K. Amero S. Nelson S. Harte P. J. Diaz M. O. (2002). A new family of cyclophilins with an RNA recognition motif that interact with members of the trx/MLL protein family in *Drosophila* and human cells. *Dev. Genes Evol.* 212 107–113. 10.1007/s00427-002-0213-8 11976948

[B2] Antonova S. V. Haffke M. Corradini E. Mikuciunas M. Low T. Y. Signor L. (2018). Chaperonin CCT checkpoint function in basal transcription factor TFIID assembly. *Nat. Struct. Mol. Biol.* 25 1119–1127. 10.1038/s41594-018-0156-z 30510221 PMC6292499

[B3] Banks C. A. S. Miah S. Adams M. K. Eubanks C. G. Thornton J. L. Florens L. Washburn M. P. (2018). Differential HDAC1/2 network analysis reveals a role for prefoldin/CCT in HDAC1/2 complex assembly. *Sci. Rep.* 8:13712. 10.1038/s41598-018-32009-w 30209338 PMC6135828

[B4] Beuchle D. Struhl G. Müller J. (2001). Polycomb group proteins and heritable silencing of *Drosophila* Hox genes. *Development* 128 993–1004. 10.1242/dev.128.6.993 11222153

[B5] Bienz M. Saari G. Tremml G. Müller J. Züst B. Lawrence P. A. (1988). Differential regulation of Ultrabithorax in two germ layers of *Drosophila*. *Cell* 53 567–576. 10.1016/0092-8674(88)90573-9 2897241

[B6] Bonnet J. Lindeboom R. G. H. Pokrovsky D. Stricker G. Çelik M. H. Rupp R. A. W. (2019). Quantification of proteins and histone marks in drosophila embryos reveals stoichiometric relationships impacting chromatin regulation. *Dev. Cell* 51 632–644.e6. 31630981 10.1016/j.devcel.2019.09.011

[B7] Brand M. Nakka K. Zhu J. Dilworth F. J. (2019). Polycomb/Trithorax antagonism: cellular memory in stem cell fate and function. *Cell Stem Cell* 24 518–533. 10.1016/j.stem.2019.03.005 30951661 PMC6866673

[B8] Breiling A. Turner B. M. Bianchi M. E. Orlando V. (2001). General transcription factors bind promoters repressed by Polycomb group proteins. *Nature* 412 651–655. 10.1038/35088090 11493924

[B9] Camasses A. Bogdanova A. Shevchenko A. Zachariae W. (2003). The CCT chaperonin promotes activation of the anaphase-promoting complex through the generation of functional Cdc20. *Mol. Cell* 12 87–100. 10.1016/S1097-2765(03)00244-2 12887895

[B10] Cao R. Wang L. Wang H. Xia L. Erdjument-Bromage H. Tempst P. (2002). Role of histone H3 lysine 27 methylation in Polycomb-group silencing. *Science* 298 1039–1043. 10.1126/science.1076997 12351676

[B11] Cavalli G. Paro R. (1999). Epigenetic inheritance of active chromatin after removal of the main transactivator. *Science* 286 955–958. 10.1126/science.286.5441.955 10542150

[B12] Celniker S. E. Keelan D. J. Lewis E. B. (1989). The molecular genetics of the bithorax complex of *Drosophila*: characterization of the products of the abdominal-B domain. *Genes Dev.* 3 1424–1436. 10.1101/gad.3.9.1424 2575066

[B13] Chen Y. (2011). *The Immunophilin FKBP39 Regulates Polycomb Group Mediated Epigenetic Control in Drosophila melanogaster*. Ph.D. thesis. Zürich: ETH Zurich. 10.3929/ethz-a-006875876

[B14] Condelli V. Crispo F. Pietrafesa M. Lettini G. Matassa D. S. Esposito F. (2019). HSP90 molecular chaperones, metabolic rewiring, and epigenetics: impact on tumor progression and perspective for anticancer therapy. *Cells* 8:532. 10.3390/cells8060532 31163702 PMC6627532

[B15] Czermin B. Melfi R. McCabe D. Seitz V. Imhof A. Pirrotta V. (2002). *Drosophila* enhancer of Zeste/ESC complexes have a histone H3 methyltransferase activity that marks chromosomal Polycomb sites. *Cell* 111 185–196. 10.1016/s0092-8674(02)00975-3 12408863

[B16] Decamillis M. Cheng N. Pierre D. Brock H. W. (1992). The polyhomeotic gene of *Drosophila* encodes a chromatin protein that shares polytene chromosome-binding sites with Polycomb. *Genes Dev.* 6 223–232. 10.1101/gad.6.2.223 1346609

[B17] Dellino G. I. Schwartz Y. B. Farkas G. McCabe D. Elgin S. C. Pirrotta V. (2004). Polycomb silencing blocks transcription initiation. *Mol. Cell* 13 887–893. 10.1016/S1097-2765(04)00128-5 15053881

[B18] Delorenzi M. Bienz M. (1990). Expression of abdominal-B homeoproteins in *Drosophila* embryos. *Development* 108 323–329. 10.1242/dev.108.2.323 1972049

[B19] Dunn A. Y. Melville M. W. Frydman J. (2001). Review: cellular substrates of the eukaryotic chaperonin TRiC/CCT. *J. Struct. Biol.* 135 176–184. 10.1006/jsbi.2001.4380 11580267

[B20] Enderle D. Beisel C. Stadler M. B. Gerstung M. Athri P. Paro R. (2011). Polycomb preferentially targets stalled promoters of coding and noncoding transcripts. *Genome Res.* 21 216–226. 10.1101/gr.114348.110 21177970 PMC3032925

[B21] Francis N. J. Saurin A. J. Shao Z. Kingston R. E. (2001). Reconstitution of a functional core Polycomb repressive complex. *Mol. Cell* 8 545–556. 10.1016/S1097-2765(01)00316-1 11583617

[B22] Gestaut D. Limatola A. Joachimiak L. Frydman J. (2019). The ATP-powered gymnastics of TRiC/CCT: an asymmetric protein folding machine with a symmetric origin story. *Curr. Opin. Struct. Biol.* 55 50–58. 10.1016/j.sbi.2019.03.002 30978594 PMC6776438

[B23] Gibson G. Gehring W. J. (1988). Head and thoracic transformations caused by ectopic expression of *Antennapedia* during *Drosophila* development. *Development* 102 657–675. 10.1242/dev.102.4.657

[B24] Green M. R. Sambrook J. (2018). Analysis and normalization of real-time polymerase chain reaction (PCR) experimental data. *Cold Spring Harb. Protoc.* 2018 769–777. 10.1101/pdb.top095000 30275081

[B25] Guenther M. G. Yu J. Kao G. D. Yen T. J. Lazar M. A. (2002). Assembly of the SMRT-histone deacetylase 3 repression complex requires the TCP-1 ring complex. *Genes Dev.* 16 3130–3135. 10.1101/gad.1037502 12502735 PMC187500

[B26] Ingham P. Whittle R. (1980). Trithorax: a new homoeotic mutation of *Drosophila melanogaster* causing transformations of abdominal and thoracic imaginal segments - I. Putative role during embryogenesis. *MGG Mol. Gen. Genet.* 179 607–614. 10.1007/BF00271751

[B27] Kaisari S. Sitry-Shevah D. Miniowitz-Shemtov S. Teichner A. Hershko A. (2017). Role of CCT chaperonin in the disassembly of mitotic checkpoint complexes. *Proc. Natl. Acad. Sci. U.S.A.* 114 956–961. 10.1073/pnas.1620451114 28096334 PMC5293070

[B28] Kassis J. A. Kennison J. A. Tamkun J. W. (2017). Polycomb and trithorax group genes in *Drosophila*. *Genetics* 206 1699–1725. 10.1534/genetics.115.185116 28778878 PMC5560782

[B29] Kim A. R. Choi K. W. (2019). TRiC/CCT chaperonins are essential for organ growth by interacting with insulin/TOR signaling in *Drosophila*. *Oncogene* 38 4739–4754. 10.1038/s41388-019-0754-1 30792539 PMC6756063

[B30] Klymenko T. Jürg M. (2004). The histone methyltransferases trithorax and ash1 prevent transcriptional silencing by Polycomb group proteins. *EMBO Rep.* 5 373–377. 10.1038/sj.embor.7400111 15031712 PMC1299022

[B31] Kuroda M. I. Kang H. De S. Kassis J. A. (2020). Dynamic competition of Polycomb and trithorax in transcriptional programming. *Annu. Rev. Biochem.* 89 235–253. 10.1146/annurev-biochem-120219-103641 31928411 PMC7311296

[B32] Laskar S. Bhattacharyya M. K. Shankar R. Bhattacharyya S. (2011). HSP90 controls SIR2 mediated gene silencing. *PLoS One* 6:e23406. 10.1371/journal.pone.0023406 21829731 PMC3150437

[B33] Lis J. (1998). Promoter-associated pausing in promoter architecture and postinitiation transcriptional regulation. *Cold Spring Harb. Symp. Quant. Biol.* 63 347–356. 10.1101/sqb.1998.63.347 10384299

[B34] Mitchison T. J. Sedat J. (1983). Localization of antigenic determinants in whole *Drosophila* embryos. *Dev. Biol.* 99 261–264. 10.1016/0012-1606(83)90275-0 6194030

[B35] Mollaaghababa R. Sipos L. Tiong S. Y. Papoulas O. Armstrong J. A. Tamkun J. W. (2001). Mutations in *Drosophila* heat shock cognate 4 are enhancers of Polycomb. *Proc. Natl. Acad. Sci. U.S.A.* 98 3958–3963. 10.1073/pnas.061497798 11274417 PMC31161

[B36] Müller J. Hart C. M. Francis N. J. Vargas M. L. Sengupta A. Wild B. (2002). Histone methyltransferase activity of a *Drosophila* Polycomb group repressor complex. *Cell* 111 197–208. 10.1016/s0092-8674(02)00976-5 12408864

[B37] Nelson C. J. Santos-Rosa H. Kouzarides T. (2006). Proline isomerization of histone H3 regulates lysine methylation and gene expression. *Cell* 126 905–916. 10.1016/j.cell.2006.07.026 16959570

[B38] Okazaki K. Kato H. Iida T. Shinmyozu K. Nakayama J. I. Murakami Y. (2018). RNAi-dependent heterochromatin assembly in fission yeast *Schizosaccharomyces pombe* requires heat-shock molecular chaperones Hsp90 and Mas5. *Epigenetics Chromatin* 11:26. 10.1186/s13072-018-0199-8 29866182 PMC5985592

[B39] Papp B. Müller J. (2006). Histone trimethylation and the maintenance of transcriptional ON and OFF states by trxG and PcG proteins. *Genes Dev.* 20 2041–2054. 10.1101/gad.388706 16882982 PMC1536056

[B40] Pattatucci A. M. Kaufman T. C. (1991). The homeotic gene sex combs reduced of *Drosophila melanogaster* is differentially regulated in the embryonic and imaginal stages of development. *Genetics* 129 443–461. 10.1093/genetics/129.2.443 1683847 PMC1204635

[B41] Plaza S. Prince F. Jaeger J. Kloter U. Flister S. Benassayag C. (2001). Molecular basis for the inhibition of *Drosophila* eye development by *Antennapedia*. *EMBO J.* 20 802–811. 10.1093/emboj/20.4.802 11179224 PMC145416

[B42] Rastelli L. Chan C. S. Pirrotta V. (1993). Related chromosome binding sites for zeste, suppressors of zeste and Polycomb group proteins in *Drosophila* and their dependence on enhancer of zeste function. *EMBO J.* 12 1513–1522. 10.1002/j.1460-2075.1993.tb05795.x 8467801 PMC413364

[B43] Riley P. D. Carroll S. B. Scott M. P. (1987). The expression and regulation of Sex combs reduced protein in *Drosophila* embryos. *Genes Dev.* 1 716–730. 10.1101/gad.1.7.716 2892760

[B44] Roobol A. Roobol J. Carden M. J. Smith M. E. Hershey J. W. Bastide A. (2014). The chaperonin CCT interacts with and mediates the correct folding and activity of three subunits of translation initiation factor eIF3: b, i and h. *Biochem. J.* 458 213–224. 10.1042/bj20130979 24320561

[B45] Sawarkar R. Paro R. (2013). Hsp90@chromatin.nucleus: an emerging hub of a networker. *Trends Cell Biol.* 23 193–201. 10.1016/j.tcb.2012.11.007 23286900

[B46] Sawarkar R. Sievers C. Paro R. (2012). Hsp90 globally targets paused RNA polymerase to regulate gene expression in response to environmental stimuli. *Cell* 49 807–818. 10.1016/j.cell.2012.02.061 22579285

[B47] Schuettengruber B. Ganapathi M. Leblanc B. Portoso M. Jaschek R. Tolhuis B. (2009). Functional anatomy of Polycomb and trithorax chromatin landscapes in *Drosophila* embryos. *PLoS Biol.* 7:e13. 10.1371/journal.pbio.1000013 19143474 PMC2621266

[B48] Schwartz Y. B. Kahn T. G. Stenberg P. Ohno K. Bourgon R. Pirrotta V. (2010). Alternative epigenetic chromatin states of Polycomb target genes. *PLoS Genet.* 6:e1000805. 10.1371/journal.pgen.1000805 20062800 PMC2799325

[B49] Simon J. Chiang A. Bender W. (1992). Ten different Polycomb group genes are required for spatial control of the abdA and AbdB homeotic products. *Development* 114 493–505. 10.1242/dev.114.2.493 1350533

[B50] Sollars V. Lu X. Xiao L. Wang X. Garfinkel M. D. Ruden D. M. (2003). Evidence for an epigenetic mechanism by which Hsp90 acts as a capacitor for morphological evolution. *Nat. Genet.* 33 70–74. 10.1038/ng1067 12483213

[B51] Sternlicht H. Farr G. W. Sternlicht M. L. Driscoll J. K. Willison K. Yaffe M. B. (1993). The t-complex polypeptide 1 complex is a chaperonin for tubulin and actin in vivo. *Proc. Natl. Acad. Sci. U.S.A.* 90 9422–9426. 10.1073/pnas.90.20.9422 8105476 PMC47580

[B52] Sun L. Liu X. M. Li W. Z. Yi Y. Y. He X. Wang Y. (2020). The molecular chaperone Hsp90 regulates heterochromatin assembly through stabilizing multiple complexes in fission yeast. *J. Cell Sci.* 133:jcs244863. 10.1242/jcs.244863 32499408

[B53] Tariq M. Nussbaumer U. Chen Y. Beisel C. Paro R. (2009). Trithorax requires Hsp90 for maintenance of active chromatin at sites of gene expression. *Proc. Natl. Acad. Sci. U.S.A.* 106 1157–1162. 10.1073/pnas.0809669106 19144915 PMC2633526

[B54] Tariq M. Saze H. Probst A. V. Lichota J. Habu Y. Paszkowski J. (2003). Erasure of CpG methylation in *Arabidopsis* alters patterns of histone H3 methylation in heterochromatin. *Proc. Natl. Acad. Sci. U.S.A.* 100 8823–8827. 12853574 10.1073/pnas.1432939100 PMC166397

[B55] Umer Z. Akhtar J. Khan M. H. F. Shaheen N. Haseeb M. A. Mazhar K. (2019). Genome-wide RNAi screen in *Drosophila* reveals Enok as a novel trithorax group regulator. *Epigenetics Chromatin* 12:55. 10.1186/s13072-019-0301-x 31547845 PMC6757429

[B56] Wang H. Wang L. Erdjument-Bromage H. Vidal M. Tempst P. Jones R. S. (2004). Role of histone H2A ubiquitination in Polycomb silencing. *Nature* 431 873–878. 10.1038/nature02985 15386022

[B57] Wang Y. J. Brock H. W. (2003). Polyhomeotic stably associates with molecular chaperones Hsc4 and Droj2 in *Drosophila* Kc1 cells. *Dev. Biol.* 262 350–360. 10.1016/S0012-1606(03)00396-8 14550797

[B58] Wells C. A. Dingus J. Hildebrandt J. D. (2006). Role of the chaperonin CCT/TRiC complex in G protein βγ-dimer assembly. *J. Biol. Chem.* 281 20221–20232. 10.1074/jbc.M602409200 16702223

[B59] Won K.-A. Schumacher R. J. Farr G. W. Horwich A. L. Reed S. I. (1998). Maturation of human cyclin E requires the function of eukaryotic chaperonin CCT. *Mol. Cell. Biol.* 18 7584–7589. 10.1128/mcb.18.12.7584 9819444 PMC109339

[B60] Zhu J. Ordway A. J. Weber L. Buddika K. Kumar J. P. (2018). Polycomb group (PcG) proteins and Pax6 cooperate to inhibit in vivo reprogramming of the developing *Drosophila* eye. *Development* 145:dev160754. 10.1242/dev.160754 29530880 PMC5963869

[B61] Zink B. Paro R. (1989). In vivo binding pattern of a trans-regulator of homoeotic genes in *Drosophila melanogaster*. *Nature* 337 468–471. 2563569 10.1038/337468a0

