# Supplementary figures and images for: Polycomb Requires Chaperonin Containing TCP-1 Subunit 7 for Maintaining Gene Silencing in Drosophila
Source: Front Cell Dev Biol. 2021 Oct 1;9:727972. doi: 10.3389/fcell.2021.727972 (PMC8517254; doi:10.3389/fcell.2021.727972)

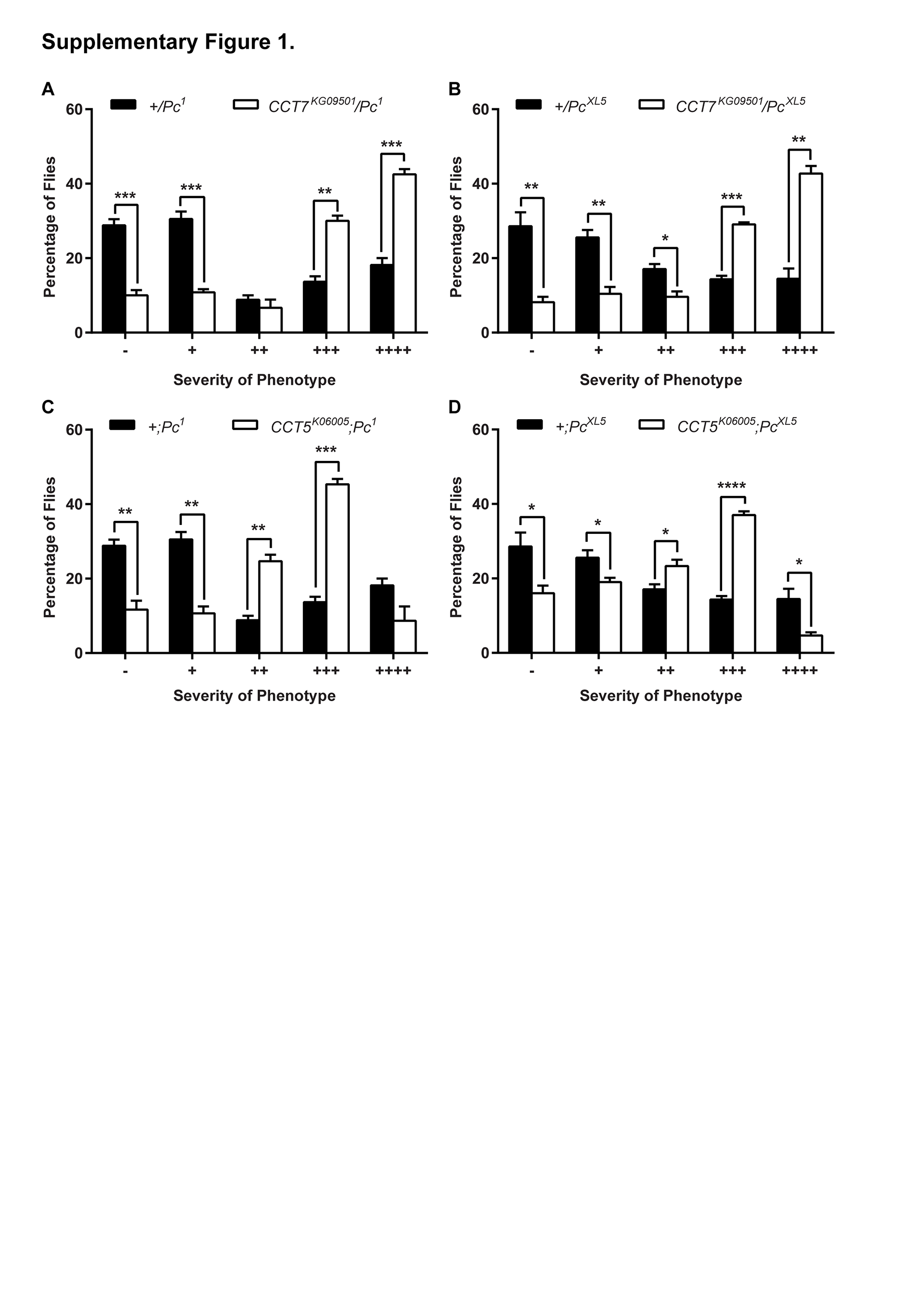

Supplement: Supplementary Figure 1 — CCT7 and CCT5 mutants enhance extra sex comb phenotype of Pc. (A–D) CCT7KG09501 and CCT5K06005 mutants were crossed to two different Pc (Pc1 and PcXL5) alleles and double mutant (CCT7/Pc and CCT5;Pc) male flies in the progeny were scored for extra sex comb phenotype. Heterozygous male flies for Pc (+/Pc) from the cross of w1118 with Pc alleles were used as control. CCT7KG09501 mutation enhanced the extra sex comb phenotype of both Pc1 and PcXL5 in double mutant CCT7KG09501/Pc1 (A) and CCT7KG09501/PcXL5 (B) as compared to control. Similarly, CCT5K06005 mutant showed increase in the extra sex comb phenotype in double mutant CCT5K06005; Pc1 (C) and CCT5K06005;PcXL5 (D) progeny as compared to control. Severity of phenotype and statistical analysis was performed as described in Figure 1. [file Image_1.tif]

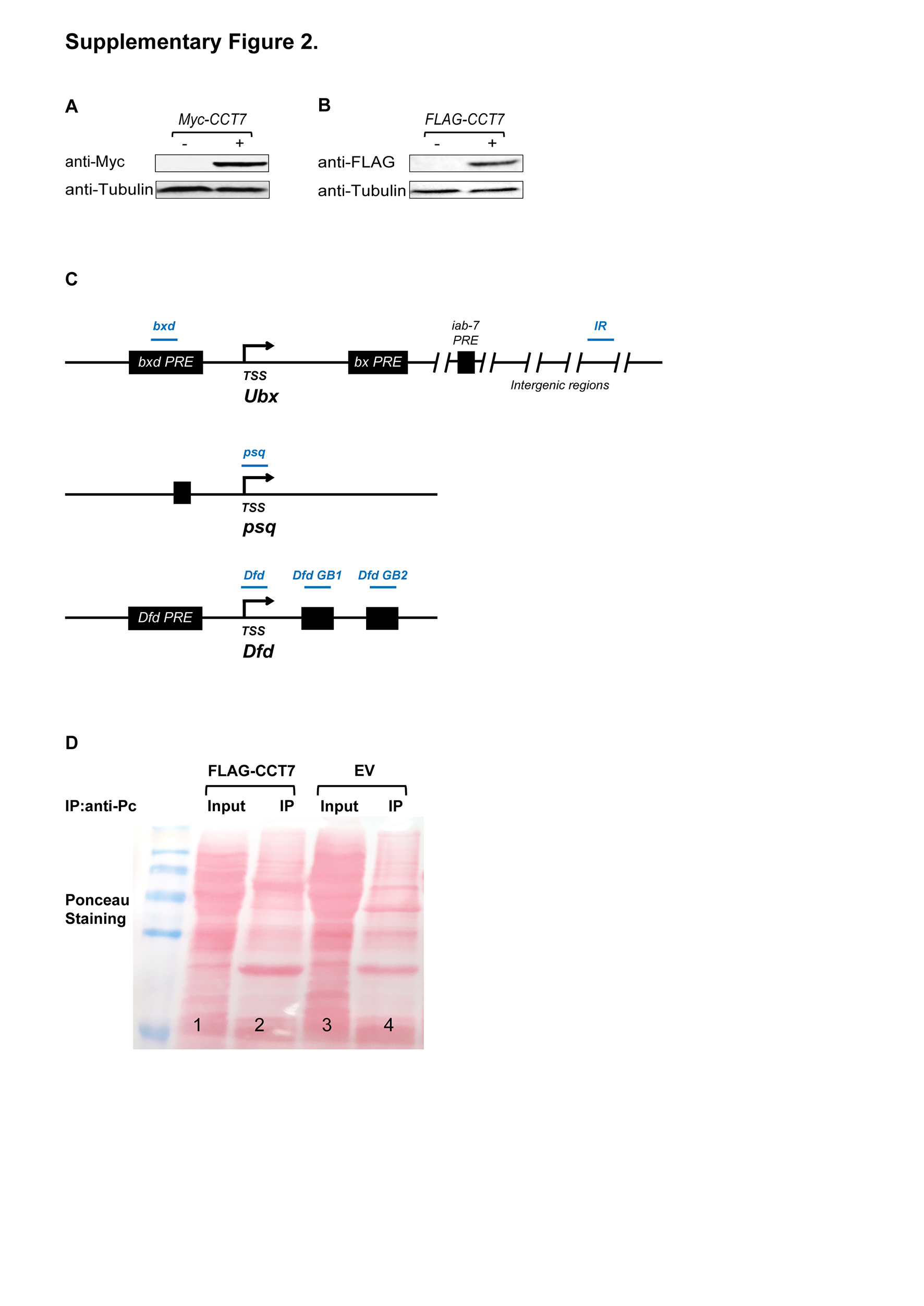

Supplement: Supplementary Figure 2 — Western blot confirms the expression of epitope-tagged CCT7 transgene. (A) Western blot showed Myc-tagged-CCT7 specifically detected in larval extracts where the expression of UAS-Myc-CCT7 was induced by crossing with pTub-GAL4 (+) driver line as compared to control (−). (B) Western blot showed the expression of FLAG-CCT7 in stable cells induced with 500μM CuSO4 (+) as compared to un-induced control (-). Tubulin was used as loading control. (C) Schematic of the DNA regions amplified using specific primers, represented as blue lines, for real time PCR analysis of ChIP DNA. (D) Ponceau stained blot corresponding to the Co-IP performed from Drosophila cells (Figure 3F). [file Image_2.tif]
