# Supplementary material for: Polycomb Requires Chaperonin Containing TCP-1 Subunit 7 for Maintaining Gene Silencing in Drosophila
Source: Front Cell Dev Biol. 2021 Oct 1;9:727972. doi: 10.3389/fcell.2021.727972 (PMC8517254; doi:10.3389/fcell.2021.727972)
Supplement: Supplementary Table 2 — List of primers used in this study. [file Table_2.docx]

**Supplementary Table 2: List of primers used in this study**

| **Sr No.** | **Primer Name** | **Sequence** | **Purpose** |  |
| --- | --- | --- | --- | --- |
| 1 | T7-LacZ F | **TAATACGACTCACTATAGGGAGA**GGAAGATCAGGATATGTGG | LacZ  dsRNA |  |
| 2 | T7-LacZ R | **TAATACGACTCACTATAGGGAGA**CTTCATCAGCAGGATATCC |  |  |
| 3 | T7-CCT7 F | **TAATACGACTCACTATAGGGAGA**GCAGCCACTGCCATGTC | CCT7  dsRNA |  |
| 4 | T7-CCT7 R | **TAATACGACTCACTATAGGGAGA**CCACCGCAAGCCTTCAT |  |  |
|  |  |  |  |  |
| 5 | CCT7 F | CACCATGCAACCGCAAATCGTGCT | Primers used for pENTR/D-TOPO  cloning |  |
| 6 | CCT7 R (NS) | CATGGGCCTGCCCATTCCG |  |  |
| 7 | CCT7 R (WS) | TTACATGGGCCTGCCCATTCCG |  |  |
|  |  |  |  |  |
| 8 | bxd-s-low | GCACTTAAAACGGCCATTACGAA | Primers used for analysis of  ChIP |  |
| 9 | bxd-s-up | GACGTGCGTAAGAGCGAGATACAG |  |  |
| 10 | Dfd F | AACTCTCCGTGCGAGCGAAC |  |  |
| 11 | Dfd R | ATGCTCCCTCTCAGTCGCGCT |  |  |
| 12 | Intergenic Region F (IR) | CCGAACATGAGACATGGAAAA |  |  |
| 13 | Intergenic Region R (IR) | AAAGTGCCGACAATGCAGTTA |  |  |
| 14 | psq_TSS_F | ATAAGGCGATGCCACCTAGTTA |  |  |
| 15 | psq_TSS_R | AATGTAGCAAAAGGTGCTCAAAG |  |  |
| 16 | Dfd_GB1_F | ACTACTTGCAAAAGCAGCGC |  |  |
| 17 | Dfd_GB1_R | GAAACTTTGGGTCCAAGCCAT |  |  |
| 18 | Dfd_GB2_F | ATGGGCTCAGTTGAGTTGAC |  |  |
| 19 | Dfd_GB2_R | TATGGTCGAACTGGAGTATC |  |  |
|  |  |  |  |  |
| 20 | Act57B F | TGTGTGACGATGAAGTTGCTGC | Primers used for qPCR Analysis |  |
| 21 | Act57B R | ATCACCGACGTACGAGTCCTT |  |  |
| 22 | CCT7_RT-F | TGATTGTGGATGCCCACGG |  |  |
| 23 | CCT7_RT-R | TGGGTGCACTCCCTCCTCC |  |  |
| 24 | AbdB ex1.1 F | CAACTACCGAACTAAGCTGC |  |  |
| 25 | AbdB ex1.2 R | CACAATGAGGAGCAAGGATG |  |  |
| 26 | Dfd F | CGATGGCGAACGGATCATCTA |  |  |
| 27 | Dfd R | GCGTCAGGTAGCGGTTGTAGTGG |  |  |
| 28 | Ubx F | ATGAACTCGTACTTTGAACAGGC |  |  |
| 29 | Ubx R | CCAGCGAGAGAGGGAATCC |  |  |
| 30 | Antp F | GCCTCCGCTGGTGGATCAAAT |  |  |
| 31 | Antp R | GCTGGTACATGCCCATGTTGTGAT |  |  |
| 32 | psq_E3F | GCAAACATCCCACAATTATCCT |  |  |
| 33 | psq_E4R | TCGCAGAGTCCCTTGATCTT |  |  |
| 34 | Pc_F | GGAGTAAGGGGAAGTTGGGGCG |  |  |
| 35 | Pc_R | CGGCGATCCAGGATGTTTAC |  |  |
