# Supplementary material for: Polycomb Requires Chaperonin Containing TCP-1 Subunit 7 for Maintaining Gene Silencing in Drosophila
Source: Front Cell Dev Biol. 2021 Oct 1;9:727972. doi: 10.3389/fcell.2021.727972 (PMC8517254; doi:10.3389/fcell.2021.727972)
Supplement: Supplementary file 5 [file Data_Sheet_1.docx]

**Supplementary data**

**Supplementary Figure Legends**

**Figure 1. *CCT7*** **and *CCT5* mutants enhance extra sex comb phenotype of *Pc*.**

**(A-D)** *CCT7^KG09501^* and *CCT5^K06005^* mutants were crossed to two different *Pc* (*Pc^1^* and *Pc^XL5^*) alleles and double mutant (*CCT7/Pc and CCT5;Pc*) male flies in the progeny were scored for extra sex comb phenotype. Heterozygous male flies for *Pc* (*+/Pc*) from the cross of *w^1118^* with *Pc* alleles were used as control. *CCT7^KG09501^* mutation enhanced the extra sex comb phenotype of both *Pc^1^* and *Pc^XL5^* in double mutant *CCT7^KG09501^/Pc^1^* **(A)** and *CCT7^KG09501^/Pc^XL5^* **(B)** as compared to control. Similarly, *CCT5^K06005^* mutant showed increase in the extra sex comb phenotype in double mutant *CCT5^K06005^;Pc^1^* **(C)** and *CCT5^K06005^;Pc^XL5^* **(D)** progeny as compared to control. Severity of phenotype and statistical analysis was performed as described in Figure 1.

**Figure 2. Western blot confirms the expression of epitope-tagged *CCT7* transgene.**

**(A)** Western blot showed Myc-tagged-CCT7 specifically detected in larval extracts where the expression of *UAS-Myc-CCT7* was induced by crossing with pTub-GAL4 (+) driver line as compared to control (-). **(B)** Western blot showed the expression of FLAG-CCT7 in stable cells induced with 500μM CuSO_4_ (+) as compared to un-induced control (-). Tubulin was used as loading control. **(C)** Schematic of the DNA regions amplified using specific primers, represented as blue lines, for real time PCR analysis of ChIP DNA. **(D)** Ponceau stained blot corresponding to the Co-IP performed from *Drosophila* cells (**Figure 3F**).

**Supplementary Tables**

**Table 1**. Percentages of extra sex comb flies scored in different categories corresponding to Figure 1A-D and Supplementary Figure 1A-D.

**Table 2.** List of primers used in this study.
